# Inferring on Joint Associations From Marginal Associations and a Reference Sample

**DOI:** 10.1002/bimj.70114

**Published:** 2026-04-13

**Authors:** Tzviel Frostig, Ruth Heller

**Affiliations:** ^1^ Department of Statistics and Operations Research Tel‐Aviv University Tel‐Aviv Israel

## Abstract

We present a method to infer on joint regression coefficients obtained from marginal regressions using a reference panel. This type of scenario is common in genetic fine‐mapping, where the estimated marginal associations are reported in genomewide association studies, and a reference panel is used for inference on the association in a joint regression model. We show that ignoring the uncertainty due to the use of a reference panel instead of the original design matrix can lead to a severe inflation of false discoveries and a lack of replicable findings. We derive the asymptotic distribution of the estimated coefficients in the joint regression model and show how it can be used to produce valid inference. We address two settings: inference within regions that are preselected as well as within regions that are selected based on the same data. By means of real data examples and simulations, we demonstrate the usefulness of our suggested methodology.

## 1 Introduction

We consider the standard linear model: a quantitative response vector $y\in R^{n}$ (n being the number of observations), which is modeled as a linear function of X, y=Xβ+ε, where $\varepsilon ∼(0,\sigma^{2}I_{n})$, X is an n×p normalized covariates matrix and p is the number of covariates. The ordinary least squares (OLS) estimate of the coefficients is $\hat{\beta }={\left(X^{\prime }X\right)}^{-1}X^{\prime }y$ for p<n. Conditioning on X, the distribution of $\hat{\beta }$ is multivariate normal: $\hat{\beta }|X∼N_{p}\left(\beta ,\sigma^{2}{\left(X^{\prime }X\right)}^{-1}\right)$. The distribution of $\hat{\beta }$ given X is exact if ε follows a normal distribution, and it holds asymptotically (in n) otherwise.

We address the setting in which $n_{o}+n_{r}$ covariate vectors are sampled independently from the same distribution, but the analyst only has full access to the $n_{r}$ covariate vectors and to summary‐level statistics using the first $n_{o}$ covariate vectors and an outcome. Throughout, we use r and o to indicate “reference” versus “original” data (so $n_{r}$ refers to the number of observations in the reference panel). We denote by $X_{o}$ and $X_{r}$ the covariate matrices obtained from the $n_{o}$ and $n_{r}$ covariate vectors. The specific information the analyst has access to is the marginal association of each of the p covariates with the outcome, ${X_{o}}^{\prime }y_{o}$, as well as the reference panel, $X_{r}$. Yang et al. (2012) suggested estimating the coefficients by plugging into the OLS estimate ${X_{r}}^{\prime }X_{r}$ for $X^{\prime }X$ and ${X_{o}}^{\prime }y_{o}$ for $X^{\prime }y$ (see Equation 1). Their suggestion has been used in many studies, including Horikoshi et al. (2016), Marouli et al. (2017), and Van Der Harst et al. (2012).

This setting is of interest in genomewide association studies (GWAS), where the goal is to find genomic regions associated with a particular phenotype (Schaid et al. 2018). In this context, the covariates are single‐nucleotide polymorphisms (SNPs), which are genetic variations at a single base‐pair position, and the outcome is a phenotype or trait of interest. Since GWAS typically involves many more SNPs than observations, each SNP association is often estimated marginally, without considering the influence of other SNPs. Marginal associations can be large even for noncausal SNPs due to linkage disequilibrium (LD) with a causal SNP. LD refers to the association of alleles at different loci in a population, indicating that certain alleles are inherited together because of their physical proximity on the chromosome (Slatkin 2008). The process of extracting candidate “causal” SNPs—where a SNP is considered “causal” if it remains associated with the phenotype after conditioning on all other SNPs—is called fine‐mapping and is a crucial step in identifying functionally relevant variants in GWAS. To extract “causal” SNPs from GWAS, the SNPs are first divided into regions of interest according to the statistical significance of their marginal association with the phenotype and their LD (Schaid et al. 2018). Identifying “causal” SNPs within a region requires approaches that account for LD and model uncertainty. One possible approach is the use of penalized regression methods, such as LASSO and elastic‐net (Tibshirani 1996; H. Zou and Hastie 2005; Vignal et al. 2011; Waldmann et al. 2013), which are applicable even when the number of observations is smaller than the number of SNPs considered in the region. These methods perform variable selection by shrinking effect sizes toward zero, typically resulting in sparse models where only a subset of correlated SNPs is retained. However, penalized regression tends to select one representative SNP from a group of correlated variants rather than all plausible “causal” SNPs. Moreover, since the methods use regularization, standard inference techniques are not applicable and post‐selection inference techniques are required to properly account for the selection step when inferring on the parameters (Lee et al. 2016; Loftus and Taylor 2015; Taylor and Tibshirani 2018). A different approach is to further divide the regions into smaller regions in which the number of SNPs considered in each such region is smaller than the number of observations (Schaid et al. 2018). The advantage over the penalized regression approaches is that standard regression methods can be applied, and the inference is more straightforward (i.e., p‐values and confidence intervals can be easily computed as long as the full data are available). Moreover, even though the inference within smaller regions is marginalized over the SNPs outside these regions, it is still preferred over complete marginalization, where the phenotype is regressed on each SNP separately. This is the approach we consider in this paper.

Although sharing $X_{o}$ between analysts is useful for fine‐mapping, it can be problematic due to (i) logistical issues since the data are large and (ii) privacy issues that come with using such sensitive data (Zhu and Stephens 2017). So it should not come as a surprise that GWAS researchers usually do not disclose their study's genetic data but only the marginal regression results. Fine‐mapping may still be performed using public genetic repositories, such as the 1000 Genomes Project, HapMap, and the UK‐biobank (Bycroft et al. 2018; 1000 Genomes Project Consortium et al. 2015). The marginal association can be transformed into approximate joint associations using the reference panel LD estimate (Yang et al. 2012).

The use of summary‐level statistics is appealing and has been extensively explored in the context of Bayesian fine‐mapping (Benner et al. 2016; Zhu and Stephens 2017; Y. Zou et al. 2022). Benner et al. (2017) observed that employing small reference panels increases the likelihood of false discoveries and recommended using larger reference panels for more reliable results. Y. Zou et al. (2022) showed via simulation that even when using a well‐matched reference panel the Bayesian fine‐mapping methods suffer from degradation. The degradation is more pronounced the smaller the reference panel is. Furthermore, there are additional difficulties in using reference panels due to the potential heterogeneity between the original study and reference panel samples. This heterogeneity may arise from various factors, including differences in imputation quality and the genotyping platforms used. This heterogeneity can result in inaccurate estimates of LD and lead to biased fine‐mapping results (Kanai et al. 2022). Therefore, it is important to carefully consider the choice of reference panel and the potential impact of sample heterogeneity when using summary‐level statistics for fine‐mapping in GWAS.

This paper investigates the properties of the commonly used frequentist estimator of β (Section 2). We show that even in the ideal scenario where both the reference and original samples are sampled from the same distribution, there is additional variance that must be accounted for when inferring on β. Failing to take into account this additional variance leads to an increase in type I errors (Section 3). To account for the variance inflation, we rely on results regarding the vectorized correlation matrix asymptotic distribution (Browne and Shapiro 1986; Neudecker and Wesselman 1990). We obtain the asymptotic distribution of the estimated joint coefficients and suggest an estimator of the additional variance (Section 3). Our analysis elucidates the conditions under which this variance inflation can be substantial or negligible.

We show that by appropriately adjusting for the fact that the reference panel, regardless of size, represents an additional random sample of individuals, one can still achieve robust and powerful inferential results (Sections 5 and 6). We address two settings. First, the region of interest is set in advance. Second, it is selected based on the data (e.g., selecting regions with strong marginal associations in the initial GWAS analysis), thus requiring adjusting for selection bias. To correct for the selection bias, we incorporate the post‐selection after aggregate tests (PSAT; Heller et al. 2019) framework, thereby enabling valid inference post‐selection (review of PSAT is given in Section 4, with simulation results in Section 5.3). We note that although the harmful effect of selection bias is well established, as far as we know, it is completely ignored in the literature on fine‐mapping. The typical analysis scans through the marginal associations in the initial GWAS, and the regions around marginal association are fine‐mapped with the assumption that such a region has at least one “causal” variant. The danger of false positives is circumvented by selecting only p‐values $<5\times \;10^{-8}$, which is a very stringent threshold. PSAT can be used with a more lenient threshold, thus it is potentially more powerful in addition to having a theoretical guarantee.

## 2 Setup and Goal

We consider the setting for a prespecified single region where the number of observations is larger than the number of covariates. In Section 4, we extend the suggested method to multiple regions that we first screen, and only regions judged to contain signal are further analyzed.

Let $X_{i,.}^{*}$ denote a covariate vector from a multivariate distribution, with $E(X_{i,.}^{*})=\mu$ (without loss of generality, we assume μ=0) and $V(X_{i,.}^{*})=\Sigma$, where E and V are the expected value and variance operators. For iid vectors $X_{i,.}^{*},i=1,\text{…},n_{r}+n_{o}$, let the rows of $X_{r}^{*}$ and $X_{o}^{*}$ consist of the first $n_{r}$ and the last $n_{o}$ vectors, respectively. Then $X_{r}$ and $X_{o}$ are the matrices resulting from the standardization of $X_{r}^{*}$ and $X_{o}^{*}$, respectively, that is, each column has a mean of 0 and standard deviation 1. We assume $y_{o}$ is also standardized. So the marginal associations are

$${\hat{\beta }}_{o}^{m}={\left(d({X_{o}}^{\prime }X_{o})\right)}^{-1}{X_{o}}^{\prime }y_{o}={n_{o}}^{-1}{X_{o}}^{\prime }y_{o},$$

where $d:R^{p}\times R^{p}\to R^{p}\times R^{p}$ is the matrix operator that sets all non‐diagonal elements of a square matrix to zero. The second equality follows since $X_{o}$ is standardized.

Our available data are the reference panel and the marginal associations from the original study, ${\hat{\beta }}_{o}^{m}$. Our goal is fine‐mapping: identifying the SNPs associated with the phenotype in a region of interest while conditioning on the remaining SNPs in the region.

The marginal association given the covariates ${\hat{\beta }}_{o}^{m}|X_{o}∼N_{p}\left({\hat{R}}_{o}\beta ,\frac{\sigma^{2}}{n_{o}}{\hat{R}}_{o}\right)$, where ${\hat{R}}_{o}={X_{o}}^{\prime }X_{o}/n_{o}$ is a consistent estimator for R, the correlation matrix of the covariate vector. ${\hat{R}}_{o}$ (or $X_{o}$) is not available to the analyst. Instead, we can use ${\hat{R}}_{r}={X_{r}}^{\prime }X_{r}/n_{r}$, which is also a consistent estimator of R. A natural estimate of β is

(1) $${\hat{\beta }}_{mc}=\frac{n_{r}}{n_{o}}{\left({X_{r}}^{\prime }X_{r}\right)}^{-1}{X_{o}}^{\prime }y_{o}={\hat{R}}_{r}^{-1}{\hat{\beta }}_{o}^{m}.$$

Yang et al. (2012) suggested estimating the distribution of ${\hat{\beta }}_{mc}$ by $N_{p}\left(\beta ,n_{o}^{-1}\sigma^{2}{\hat{R}}_{r}^{-1}\right)$, but this approximation ignores the fact that $X_{o}$ and $X_{r}$ are not identical. Our derivation of the asymptotic distribution of ${\hat{\beta }}_{mc}$ in Theorem 3.1 suggests that, even when $X_{r}^{*}$ and $X_{o}^{*}$ are sampled independently from the same population, using the naive approximation of the coefficients' distribution to test the null hypothesis that the coefficient is zero results in a failure to control the probability of false positives (FP).

Using the above approximate distribution of ${\hat{\beta }}_{mc}$ for inference is henceforth referred to as the *naive* approach. It leads to an underestimation of the ${\hat{\beta }}_{mc}$ variance and an increase in FP. This causes a lack of replicability for studies based on summary‐level statistics. For example, the FP are almost 50% of the discoveries in some of the settings in Figure 2A. The additional variance must be taken into consideration for a valid inference on β. Our goal is to quantify this variance and propose useful, asymptotically valid estimates for powerful testing procedures.

**FIGURE 1:**
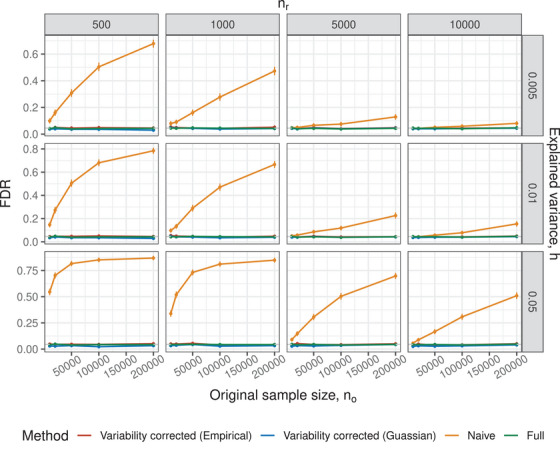
The FDR versus $n_{o}$ in settings with simulated Gaussian covariates. Each plot in the facet is a combination of the reference sample size $n_{r}$ (columns) and explained variance h (rows, Equation 6). Vertical lines are the 2 standard errors around the estimate, and the gray horizontal line is the nominal FDR level of 0.05.

**FIGURE 2:**
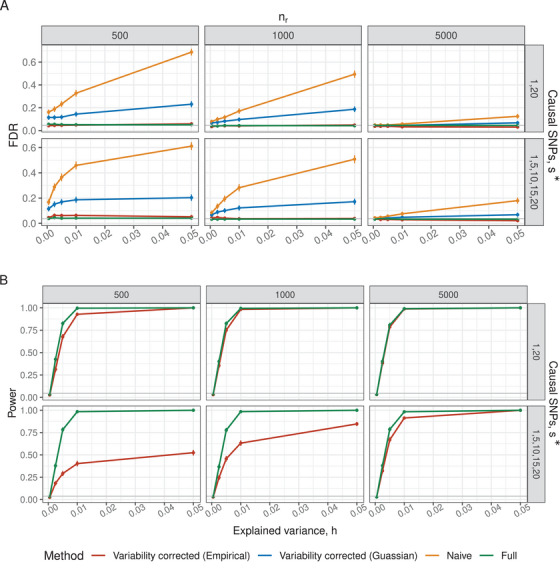
The FDR (A) and power (B) versus h (defined in Equation 6) in settings mimicking genomic covariates. Each plot in the facet is a combination of “causal” SNP indices, $s^{*}$ (rows), and the reference sample size, $n_{r}$ (columns). The number of observations in the original study is $n_{o}=10^{4}$, and the distribution of covariates is kept constant across all scenarios (see the text for details). Vertical lines are the two standard errors around the estimate, and the gray horizontal line is the nominal FDR level 0.05.

## 3 Inferring the Joint Effects Without Selection

### 3.1 Distribution of Estimated Coefficients Using a Reference Panel

In order to infer on β, we derive the asymptotic distribution of ${\hat{\beta }}_{mc}$, given in Theorem 3.1. The theorem relies on obtaining the covariance matrix of the vectorized estimated correlation matrix (Neudecker and Wesselman 1990), see Theorem S1.1. If $\hat{R}$ is an estimate of R based on n observations then $\lim_{n\to \infty }\sqrt{n}\;vec\left(\hat{R}-R\right)\to N_{p^{2}}\left(0,V_{\hat{R}}\right)$, where $vec:R^{p\times p}\to R^{p^{2}}$ is the vectorization operator, which concatenates the p columns of the square matrix to a vector. Thus, $V_{\hat{R}}$ is the variance of the limiting distribution of the vectorized estimated correlation matrix.

Theorem 3.1 Assume the following for $X_{i,.}^{*}\in R^{p}$: $V(X_{i,.}^{*})=\Sigma$ is of full rank; a bounded fourth moment, $\forall j\;E(X_{i,j}^{*4})<\infty$. Assume also that $X_{o},X_{r}$ are the matrices with standardized columns of the identically distributed rows $X_{i,.},i=1,\text{…},n_{o}+n_{r}$, with correlation matrix R. The asymptotic distribution of ${\hat{\beta }}_{mc}$ as $\lim_{n_{r},n_{o}\to \infty }n_{r}/n_{o}\to c\geq 0$ is

(2) $$\begin{array}{ccc} & & \lim_{n_{r},n_{o}\to \infty }\sqrt{n_{r}}\left({\hat{\beta }}_{mc}-\beta \right)∼N_{p}(0,c\sigma^{2}R^{-1}\\ & & \;+\left(1+c\right)\left(\beta^{\prime }⊗R^{-1}\right)V_{\hat{R}}\left(\beta ⊗R^{-1}\right)), \end{array}$$

where ⊗ is the Kronecker product.

Let $\Sigma_{mc}=V\left({\hat{\beta }}_{mc}\right)$, and ${\hat{\Sigma }}_{mc}$ denote its estimator. Examination of the asymptotic covariance provides the following insights. First, if the vector β=0, then using the reference panel does not increase the asymptotic variance of the estimator, implying that the naive approximation of ${\hat{\beta }}_{mc}$ is correct under the global‐null hypothesis of no association between all p variables and the response. Second, the variance expression contradicts the claim in Benner et al. (2017) that large reference panels can eliminate the problem. The primary issue addressed is the discrepancy between the actual variance of the estimator (Equation 2) and the *naive* variance estimator. The *naive* variance estimator scales with $O(n_{o}^{-1})$, whereas the actual variance scales in $O(n_{o}^{-1}+n_{r}^{-1})$. This implies that the *naive* variance estimator can seriously underestimate the variance, leading to an inflation in type I error that depends on the ratio c. The variance of the approximated ${\hat{\beta }}_{mc}$ is

$$\frac{\sigma^{2}}{n_{o}}R^{-1}+\left(n_{o}^{-1}+n_{r}^{-1}\right)\left(\beta^{\prime }⊗R^{-1}\right)V_{\hat{R}}\left(\beta ⊗R^{-1}\right).$$

Therefore, even for large $n_{r}$, the *naive* variance estimator may still underestimate the variance. The first term corresponds to the variance as estimated by the *naive* variance estimator. The second term is due to not conditioning on X, thus there is additional variance that we need to account for. Tarpey et al. (2014) analyzed a related phenomenon, where when pooling samples to estimate the slope estimator, the variance is larger compared to using a single sample.

### 3.2 Estimating $\Sigma_{mc}$

In order to estimate $\Sigma_{mc}$, the parameters are replaced by their respective estimators. So, R is replaced by ${\hat{R}}_{r}$, and c is replaced by $n_{r}/n_{o}$. We proceed to describe the methods to estimate the remaining parameters: $V_{\hat{R}}$, β, and $\sigma^{2}$. For β, we will use a rough estimate to get a conservative estimate of $\Sigma_{mc}$, which will ultimately lead to a valid inference for β.

Neudecker and Wesselman (1990) gave an expression for the variance of the vectorized correlation matrix, $V_{\hat{R}}$, which depends on the variance of the vectorized estimated covariance matrix, $V_{\hat{\Sigma }}=V\left(vec(\hat{\Sigma })\right)$. There are two methods to estimate $V_{\hat{\Sigma }}$ depending on the knowledge of the distribution of $X_{i,.}^{*}$. If $X_{i,.}^{*}$ follows a Gaussian distribution $V_{\hat{\Sigma }}$ can be estimated as ${\hat{V}}_{{\hat{\Sigma }}_{r}}=2M_{s}\left({\hat{\Sigma }}_{r}⊗{\hat{\Sigma }}_{r}\right)$, where ${\hat{\Sigma }}_{r}$, is the empirical covariance matrix of the $n_{r}$ covariate vectors in the reference sample, and $M_{s}$ is defined in Theorem S1.1. In the case where the $X_{i,.}^{*}$ does not follow a Gaussian distribution, the parameter, $V_{{\hat{\Sigma }}_{r}}$, is estimated directly. Denote the estimated variance based on observation i by ${\hat{\Sigma }}_{r_{i}}=\left(X_{r_{i,.}}^{*}\right){\left(X_{r_{i,.}}^{*}\right)}^{\prime }$. The variance of the covariance matrix is estimated by

$${\hat{V}}_{{\hat{\Sigma }}_{r}}=n_{r}^{-1}\sum_{i=1}^{n_{r}}\left(vec\left({\hat{\Sigma }}_{r_{i}}\right)-vec\left({\hat{\Sigma }}_{r}\right)\right){\left(vec\left({\hat{\Sigma }}_{r_{i}}\right)-vec\left({\hat{\Sigma }}_{r}\right)\right)}^{\prime }.$$

Computing $V_{{\hat{\Sigma }}_{r}}$ involves computing the outer product of $n_{r}$ vectors of length $p^{2}$, resulting in a complexity of $O(n_{r}p^{4})$. Computing $V_{{\hat{\Sigma }}_{r}}$ for covariates from the multivariate normal distribution is only $O(p^{4})$ (see Section S2 for further details).

Since y is standardized, a conservative estimate for $\sigma^{2}$ is ${\hat{\sigma }}^{2}=1$. However, for small $\sigma^{2}$ this would result in a loss of power. Since $\sigma^{2}=1-var\left(X\beta \right)=1-\beta^{\prime }R\beta$, a natural estimator is

(3) $${\hat{\sigma }}^{2}=1-{\hat{\beta }}_{mc}{\hat{R}}_{r}{\hat{\beta }}_{mc},$$

which is asymptotically unbiased. Unless stated otherwise, we use the latter estimate in our simulations and data analyses.

The vector of coefficients of interest, β, is central to the formulation of $\Sigma_{mc}$. Notably, the vector β is unknown and represents the primary target of our inferential procedures. The point estimate, ${\hat{\beta }}_{mc}$, is a natural candidate. Assuming certain entries of β to be zero can notably reduce the computational load when calculating ${\hat{\Sigma }}_{mc}$, as detailed in Section S2. We denote the estimator that sets specific entries to zero based on a predefined threshold as the *threshold estimator*.

To derive the threshold estimator, we propose a heuristic where each coefficient $\beta_{i}$ is evaluated under the global‐null hypothesis (i.e., the vector β=0). In this scenario, the variance of the coefficients can be effectively approximated using the *naive* variance estimator. The corresponding test statistic, utilizing the naive variance estimator of ${\hat{\beta }}_{mc}$, is denoted as $T_{i}^{*}=\frac{\sqrt{n_{o}}}{\hat{\sigma }}\sqrt{d{\left({\hat{R}}_{r}^{-1}\right)}_{i,i}}{\left({\hat{\beta }}_{mc}\right)}_{i}$, where $d({\hat{R}}_{r}^{-1})$ is the main diagonal of ${\hat{R}}_{r}^{-1}$. The fundamental challenge of this heuristic is selecting an appropriate threshold for setting ${\hat{\beta }}_{i}=0$ in the estimation of $\Sigma_{mc}$.

We propose using a threshold that corresponds to the same significance level that would be used when testing $\beta_{i}$ while taking into account the uncertainty caused by estimating the correlation matrix using a reference sample. Since $T_{i}^{*}$ uses the *naive* variance estimator, it will tend to be liberal; thus, for the same significance level, if the null is rejected according to a test based on Equation (2), it will also be rejected according to the test based on $T_{i}^{*}$.

The primary limitation of this heuristic arises when most entries of β are nonzero. In such cases, applying the threshold version of ${\hat{\beta }}_{mc}$ may result in underestimating $\Sigma_{mc}$ for entries where $\beta_{i}=0$, thereby increasing the probability of a type I error. If the analyst suspects such a scenario, they can either forgo the heuristic altogether, which would result in increased computation time, or opt for a more liberal significance level to reduce the number of coefficients thresholded to zero. In our simulations, even with as many as 25% nonzero entries in ${\hat{\beta }}_{mc}$, the inference remains valid. Details are provided in Section 5.2 .

## 4 Inferring the Joint Effects Following Selection

### 4.1 Post‐Selection Inference After Aggregate Tests

The fine‐mapping goal is to identify SNPs that are significantly correlated with a specific phenotype, even after adjustment for neighboring SNPs. To accomplish this goal, an analysis of the marginal association of the SNP with the phenotype can be carried out first, and SNPs are only examined jointly in regions around a SNP with a strong marginal association with the phenotype (i.e., if the strength of the marginal association with the phenotype is above a certain threshold). Following region selection, a fine‐mapping analysis is carried out in order to identify the specific SNPs associated with the phenotype in a joint analysis. If the selection and follow‐up identification are carried out on the same data, the selection process must be accounted for to guarantee valid inference.

The approach of adjusting for selection by conditioning on the selection event has been studied extensively in recent years (Lee et al. 2016; Loftus and Taylor 2015; Reid et al. 2018; Taylor et al. 2014). Heller et al. (2019) suggested a method for inferring individual parameters (SNPs in our scenario), following the rejection of the global‐null hypothesis with a linear or quadratic aggregate test. Next, we review their PSAT method and show how to adjust for selection when only $X_{r}$ and ${X_{o}}^{\prime }y$ are known.

A possible fine‐mapping strategy is to select regions for further analysis if the marginal associations (in absolute value) in the initial GWAS analysis in that region are large enough. Specifically, if the absolute marginal coefficient estimate, $|{\hat{\beta }}_{o}^{m}|$, is above a certain threshold. Since in the suggested use, case the original reference is not available, the selection must be taken into consideration while using only the reference sample. First, the selection event is described in terms of ${\hat{\beta }}_{mc}$ and ${\hat{R}}_{r}$,

(4) $$S\left({\hat{\beta }}_{mc},{\hat{R}}_{r}\right)={\hat{\beta }}_{mc}^{\prime }{\hat{R}}_{r}e^{*}e^{{*}^{\prime }}{\hat{R}}_{r}{\hat{\beta }}_{mc}>t,$$

where $e^{*}$ is a vector of length p with entry 1 in the respective SNP index and 0 elsewhere. At this point, the PSAT method can be applied to infer on any linear combination of the coefficients, $\eta^{\prime }{\hat{\beta }}_{mc}$. PSAT requires additional conditioning on $W=\left(I-c\eta^{\prime }\right)\hat{\beta }$, where $c={\left(\eta^{\prime }\Sigma_{mc}\eta \right)}^{-1}\Sigma_{mc}\eta$. The resulting distribution is

(5) $$\eta^{\prime }{\hat{\beta }}_{mc}|S>t,W,X∼TN\left(\eta^{\prime }\beta ,\eta^{\prime }\Sigma_{mc}\eta ,A\left(W\right)\right),$$

where TN(a,b,d) denotes the truncated normal distribution with expected value of a, variance b, and truncation region of d. Since $\Sigma_{mc}$ is unknown, it needs to be estimated, as detailed next in Section 4.2. The truncation region, A(W), is given in Lemma A.1 of Heller et al. (2019). While the test statistic described in Equation (4) is quadratic (in ${\hat{\beta }}_{mc}$), PSAT can also be used when the selection is based on a linear test statistic. An implementation of the method can be found at github.com/tfrostig/PSAT.

Remark 4.1 When the global null hypothesis holds (i.e., the vector β=0), the naive approximation of the distribution of ${\hat{\beta }}_{mc}$ is valid. This means that computing any global‐null test statistic, which relies on the variance of ${\hat{\beta }}_{mc}$, becomes computationally straightforward. Therefore, in the application of PSAT using summary‐level statistics and a reference panel, the more expensive estimation of the covariance matrix, $\Sigma_{mc}$, is only performed in regions where the marginal association (which is one common example of a region‐based summary) was sufficiently strong to justify fine‐mapping of the surrounding region. This approach enhances efficiency by focusing computational resources on the regions that show evidence of association.

### 4.2 Estimating $\Sigma_{mc}$ After Selection

Following selection, we also need to adjust our procedure for estimating $\Sigma_{mc}$. Recall, there are three components that need to be estimated in order to get an estimation of $\Sigma_{mc}$: $\beta ,\sigma^{2}$, and $V_{\hat{R}}$. Estimation of the vectorized correlation matrix, $V_{\hat{R}}$, does not change, but the estimates of $\sigma^{2}$ and β do. Due to selection, $|{\hat{\beta }}_{mc}|$ is biased upwards, and this could lead to a bias in the estimation of $\Sigma_{mc}$.

According to Equation (5), the linear combination follows a truncated normal distribution, so a conditional maximum likelihood estimate (MLE) can be obtained, ${\tilde{\beta }}_{mc}=\arg\max_{\beta }\left\{l\left(\beta \right)-\log P\left(S>t\right)\right\}$, where l is the log‐likelihood for β (see Section 4.1 in Heller et al. 2019 for further details).

The MLE is unbiased after selection, thus replacing ${\hat{\beta }}_{mc}$ with ${\tilde{\beta }}_{mc}$ in the estimates of $\Sigma_{mc}$ and $\sigma^{2}$ reduces their bias. To threshold ${\tilde{\beta }}_{mc}$, we apply the same process as described in Section 3.2, replacing the estimates of β with the selection‐adjusted estimators, ${\tilde{\beta }}_{mc}$.

## 5 Simulation Studies

We use simulations in order to assess the performance of our variability corrected approach using only summary‐level data. Our goals were threefold: (1) to assess the power loss incurred from using only summary‐level data and a reference panel; (2) to assess the validity of the approach for finite samples; (3) to compare the most generally applicable variability correction, which is computationally demanding, with the variability correction method which is theoretically justified only for Gaussian covariates.

We compare the performance of four methods to infer on β:

1. *Full, serves as oracle, as it is not feasible* $X_{o}$ *when* $X_{o}$ *is not observed. Assume* $X_{o},y_{o}$ are given, and the standard OLS approach is employed. The variance estimate, therefore, is ${\hat{\sigma }}^{2}/n_{o}{\hat{R}}_{o}^{-1}$.
2. *The naive* method does not take into account the additional variance caused by $X_{r}$. The variance estimator is ${\hat{\sigma }}^{2}/n_{o}{\hat{R}}_{r}^{-1}$.
3. *Variability corrected (empirical)* based on Theorem 3.1, where $var\left(vec({\hat{\Sigma }}_{r})\right)$ is estimated empirically (see Section 3.2).
4. *Variability corrected (Gaussian)* $X_{r},X_{o}$ are assumed to follow a Gaussian distribution, and, therefore, the estimate of the variance of the vectorized sample covariance matrix, $var\left(vec\left({\hat{\Sigma }}_{r}\right)\right)$ is $2M_{s}\left({\hat{\Sigma }}_{r}⊗{\hat{\Sigma }}_{r}\right)$.

We compare a wide range of settings, first we begin by examining the case where the covariates follow a Gaussian distribution (Section 5.1), then we move to the setting where the covariates are sampled from a multinomial distribution mimicking genomic covariates, without adjustment to selection (Section 5.2), and when using PSAT to adjust for selection (Section 5.3). Furthermore, there are additional simulations and examples in the Supporting Information using actual genotypes: in Section S4.2 data from the 1000Genome reference panel is used, in Section S4.3, we examine the effects of SNPs in Chromosome‐19 on body mass index (BMI) using the UK‐biobank data; in Section S4.4, we use data from the Dallas Heart Study.

Within a simulated region, we denote by s the set of significant SNPs, and by $s^{*}$ the set of SNPs with effect. The false discovery proportion (FDP) and true discovery proportion (TDP) are

$$FDP=\frac{|s\setminus s^{*}|}{\max(|s|,1)},\;TDP=|s\cap s^{*}|/\max(1,|s^{*}|).$$

The average of the FDP and TDP across simulation serves as an estimate of the FDR and power, respectively. In all simulations, the Benjamini–Hochberg (BH) method (Benjamini and Hochberg 1995) is used to control the FDR at level 0.05. Unless stated otherwise, the number of repetitions in each simulation is 1000.

The coefficients, β, are 1, where indices match $s^{*}$ and 0 elsewhere. The fraction of explained variance, that is, the heritability of the phenotype, is denoted by

(6) $$h=\frac{\beta^{\prime }R\beta }{\sigma^{2}+\beta^{\prime }R\beta }.$$

We set h by adjusting $\sigma^{2}$.

Briefly, our key findings are as follows. First, as expected, the *Naive* estimator fails to maintain the nominal FDR level, especially when $n_{r}/n_{o}$ or h are large. Only when $n_{r}/n_{o}$ is small, and both $n_{r}$ and $n_{o}$ are sufficiently large, the FDR of the *Naive* method approaches the expected level. Second, the *Variability corrected (Empirical)* method is valid in all scenarios, meaning it provides an FDR level below the nominal level. In contrast, the *Variability corrected (Gaussian)* method is only valid when the distribution of X is Gaussian. Third, if $n_{r}$ is large enough and the signal is sparse (i.e., there are only a few SNPs/features with nonzero coefficients), the power of *Variability corrected (Empirical)* is on par with the power of the *Full* method.

### 5.1 Simulations With Gaussian‐Distributed Covariates

We evaluate the performance of different methods for inferring β in Gaussian distributed data. Specifically, we sample $X_{o}^{*},X_{r}^{*}$ from N(0,Σ), where $\Sigma_{i,j}=\rho^{\left|i-j\right|}$, ρ=0.8, the number of features considered is 20, and $s^{*}=\left\{1,20\right\}$. The number of observations in the original study and in the reference panel vary: $n_{o}\in \left\{10^{4},2\times \;10^{4},10^{5},2\times 10^{5}\right\}$; $n_{r}\in \left\{5\times \;10^{2},10^{3},5\times 10^{3},10^{4}\right\}$. We consider three values of the fraction of explained variance h∈{0.005,0.01,0.05}. $X_{o}^{*}$ and $X_{r}^{*}$ are standardized to obtain $X_{o}$ and $X_{r}$, respectively.

Figure 1 shows that the *Naive* method fails to control the FDR level at the nominal level in most scenarios considered, even when a large number of observations are available in the reference sample ($n_{r}\geq 5000$). Only when both $n_{r}$ is large and h is small, the *Naive* method FDR approaches the nominal FDR. This behavior is expected because the *Naive* method does not account for the variability introduced by estimating β using a reference sample.

In contrast, both *Variability corrected (Empirical)* and *Variability corrected (Gaussian)* maintain the FDR at the desired level across all scenarios. This is because these methods account for the variability in estimating β caused by the reference sample. Therefore, these methods can be more reliable in controlling the FDR compared to the *Naive* method, particularly in scenarios with large effect sizes or limited sample sizes.

The power of the *Variability‐corrected (Empirical)* method and the *Variability corrected (Gaussian)* is similar in most scenarios. Results are deferred to the Supporting Information due to space limitations. The *Full* method is more powerful when both $n_{o}$ and $n_{r}$ are small, but as they increase the methods perform similarly (see Figure S2).

### 5.2 A Setting Mimicking Genomic Covariates

We evaluate the performance of different methods for inferring β for simulated genomic covariates. The genotypes are generated as follows: first, we sample $X_{o}^{*},X_{r}^{*}$ as described in the previous section from N(0,Σ), with $\Sigma_{i,j}=0.95^{\left|i-j\right|}$. Second, we sample p minor allele frequencies (MAFs, one for each SNP) from Beta(1,2), then divide by two, and truncated at 0.05.

To transform Gaussian samples into SNP‐type data, we apply the function g(·;q):R→{0,1,2} to each column (of $X_{r}^{*}$ or $X_{o}^{*}$). The function g(w,q) transforms the Gaussian samples w into genotypes with the following number of minor allele copies,

$$g\left(w,q\right)=\left\{\begin{array}{cc} 0 & \text{if}\;w\leq z_{1-\frac{2}{3}q},\\ 1 & \text{if}\;z_{1-\frac{2}{3}q}<w<z_{1-\frac{1}{3}q},\\ 2 & \text{if}\;w\geq z_{1-\frac{1}{3}q}, \end{array}\right.$$

where $z_{q}$ is the qth quantile of the standard normal distribution. So the expected proportion of minor alleles copies is, i.e., the MAF is q. The function is applied to each column of $X_{r}^{*}$ and $X_{o}^{*}$. Finally, after normalizing the resulting columns, we obtain $X_{o}$ and $X_{r}$.

We present the results for $s^{*}\in \left\{\left\{1,20\right\},\left\{1,5,10,15,20\right\}\right\}$, $n_{o}=10^{4}$, and $h\in \{5\times \;10^{-4},0.0025,0.01,0.05\}$. We experimented with other parameters, but they were omitted for brevity, since the qualitative conclusions were similar.

In Figure 2A, the *Variability‐corrected (Empirical)* method controls the nominal FDR across all scenarios and for all values of $n_{r}$. The *Naive* method fails to control the nominal FDR even when $n_{r}=5000$. As h increases, the coefficient's magnitude compared to $\sigma^{2}$ increases, leading to more unaccounted variance resulting in an increase in FDR of the *Naive* method. We can also see that the *Variability‐corrected (Gaussian)* FDR decreases as $n_{r}$ increases. However, it fails to maintain the FDR unless h is small and $n_{r}=5000$. Therefore, the *Naive* and *Variability‐corrected (Gaussian)* methods are omitted from the subsequent power analysis.

In Figure 2B, the power of the methods is compared. A challenging scenario for the *Variability‐corrected (Empirical)* method is $s^{*}=\left\{1,5,10,15,20\right\}$. As the number of correlated non‐null SNPs increases, the value of |β| decreases for the same h. In addition, the additional variance due to the reference panel (i.e., $\left(\beta^{\prime }⊗R^{-1}\right)V_{\hat{R}}\left(\beta ⊗R^{-1}\right))$) is affected. In this dense setting, the difference in power between the *Full* and *Variability‐corrected (Empirical)* methods increases substantially as $n_{r}$ decreases.

When $n_{r}$ is sufficiently large, the power curves merge. This happens because the additional variance shrinks as $n_{r}$ increases (see Equation 2). For sparse signal, $s^{*}=\left\{1,20\right\}$, $n_{r}=500$ is sufficient. For denser signals where the SNPs are correlated, $n_{r}$ has to be large for adequate power.

### 5.3 A Setting Mimicking Genomic Covariates With Selection

In many applications of fine‐mapping, the initial GWAS analysis identifies at least one SNP marginally associated with a trait, for example, p‐value $<5\times \;10^{-8}$ (Schaid et al. 2018). We refer to such an analysis as marginal screening. For SNPs that pass the marginal association threshold, fine‐mapping is performed by considering the region surrounding them.

We focus on selection by marginal screening because it is the simplest and most commonly used variable selection procedure, providing a good balance between a feasible marginal analysis and a non‐feasible full joint analysis. Since conditioning on additional variables typically increases power loss in valid post‐selection inference, stepwise methods are expected to suffer from greater power loss compared with selection by marginal statistics. Accordingly, we concentrated on marginal screening in the numerical experiments of PSAT. Moreover, because forward‐stepwise selection is widely used for fine‐mapping (Yang et al. 2012), we also evaluate the performance of the conditional forward‐stepwise regression suggested in that work when using a reference panel. Our Supporting Information (Section S4.5) shows that inference following the approach in Yang et al. (2012) can inflate false positives due to a naive approximation of the variance inflation caused by the reference panel.

To mimic the setting of fine‐mapping following marginal screening, we employ the same simulation strategy as detailed at the beginning of Section 5.2. Henceforth, we demonstrate the fine‐mapping results following marginal screening for the SNP located in index 10. The region is selected if the SNP's estimated marginal coefficient is above the threshold of $\sqrt{n_{o}}\times Z_{1-0.05/20,000}$ (corresponding to testing the marginal coefficient if there is no signal nor LD at α=0.05 and applying the Bonferroni correction for 20,000 hypotheses). It imitates a GWAS where 20,000 loci are being tested marginally using a Bonferroni correction.

At each iteration, the noise, ε, is re‐sampled until the selection event occurs. All other simulation configurations are identical to those detailed in Section 5.2.

The adjustment for selection is done using PSAT as described in Section 4. Incorporating the PSAT procedure leads us to consider an alternative estimate of $\Sigma_{mc}$, and the resulting method is called *Variability corrected (MLE)*. This method differs from the *Variability‐corrected (Empirical)* approach in that the conditional MLE of β—which accounts for selection—is used to estimate $\Sigma_{mc}$ (see Section 4.2). We compare the methods with and without selection adjustment to the *Full* method. Note that selection according to marginal screening is consistent between methods, as they all rely on ${X_{o}}^{\prime }y$ (see Section S3). This comparison highlights the gap in post‐selection inference when $X_{o}$ is unavailable.

**FIGURE 3:**
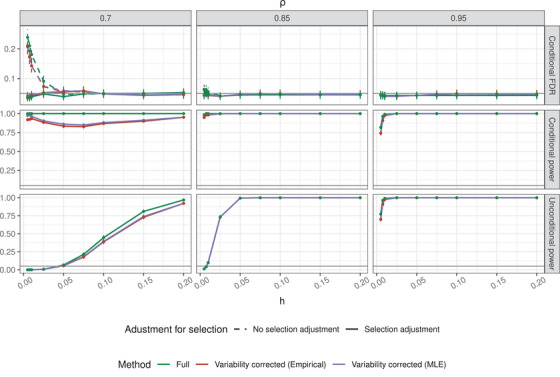
The conditional FDR, conditional power, and unconditional power as a function of h (Equation 6), in settings mimicking genomic covariates, with variability correction and PSAT. The rows indicate the measure, the columns the correlation parameter ρ. $s^{*}=\left\{1,20\right\}$, and the screening is based on the SNP located at index 10. The number of observations in the reference panel, $n_{r}=1000$. Vertical lines are the 2 standard errors around the estimate, and the gray horizontal line is the nominal FDR level of 0.05.

We consider the scenarios where $s^{*}=\left\{1,20\right\}$, $n_{r}=1000$, h∈{0.005,0.01,0.025,0.05,0.075,0.1,0.15,0.2}, and ρ∈{0.7,0.95}. In Figure 3 (top row), the conditional FDR (i.e., FDR given that the region was selected) for all methods is presented. The FDR inflation observed in methods that do not adjust for selection in the setting with ρ=0.7 is absent for ρ=0.95. This occurs because the probability of selection increases with ρ: for a region to be selected, the marginal association of the SNP located at index 10 must exceed a certain threshold. As the correlation increases, this event becomes more likely (since this SNP is more correlated with SNPs in $s^{*}$). When the selection event occurs with probability 1, no adjustment is required, and the curves for the selection‐corrected methods and the non‐adjusted method merge. The PSAT procedures that adjust for selection all maintain FDR control at the nominal level.

We consider conditional and unconditional power. The conditional power is the power to detect SNPs given that a selection event happened (only relying on iterations where the estimated marginal coefficient of the SNP located in index 10 is above the threshold). For the unconditional power, we consider the number of times the region was selected and the “causal” SNPs were detected (relying on all of the iterations).

The unconditional power increases as ρ increases. The increase in power is due to the selection event being more likely. As the correlation between the SNP located at index 10 and the “causal” SNPs increases, the selection event becomes more likely, increasing the unconditional power.

In the conditional power plot (Figure 3, middle row), we see an interesting phenomenon when ρ=0.7. The conditional power curve of the selection adjusted procedure dips before continuing to rise as h increases. The dip in power occurs because when h is small, the selection event occurs almost entirely at random (as evident by the unconditional power plots, right column). Furthermore, since ρ=0.7, the “causal” SNPs are barely correlated with the SNP located at index 10. Therefore, when the region is selected by chance, the adjustment required for the “causal” SNPs is very lenient. When h increases, the selection event is driven by the “causal” SNPs; therefore, when testing them individually, the adjustment for the SNPs driving the selection is stricter. Finally, the *Variability corrected (MLE)* is at least as powerful as the *Variance‐corrected (Empirical)* method, which is why we recommend to use it.

## 6 Association of BMI With SNPs in the FTO Gene

To assess the usefulness of the suggested method on real genomic data, we used the UK‐biobank data on the FTO gene located in Chromosome 16 (application no. 42111) in this Section. Another real data‐data example with adjustment to selection is given using Dallas Heart Study (see Section S4.4). The FTO gene was found to be correlated with obesity according to several studies (Frayling et al. 2007; Gerken et al. 2007). We used the GIANT consortium data to find SNPs with a marginal association *p*‐value of less than $5\times 10^{-8}$ with BMI and included all genotyped SNPs within a distance less than 100 kb from them. For simplicity, we did not consider imputed SNPs in this analysis. In pairs of SNPs with an absolute correlation higher than 0.97, one was randomly chosen. Overall, 33 SNPs from the FTO gene were included in the simulation.

**TABLE 1:** Results of FTO gene association with BMI.

	% (Not rejected by “Oracle” and rejected by the method)			% (Rejected by “Oracle” and rejected by the method)		
$n_{r}$	Naive	Variability corrected	Full	Naive	Variability corrected	Full
500	0.169	0.013	0.036	0.598	0.086	0.512
1500	0.085	0.014	0.033	0.540	0.206	0.513
5000	0.052	0.024	0.035	0.517	0.366	0.509

*Note:* Rows indicate the number of samples in the reference sample. The first column is the proportion of SNPs found to be associated with BMI according to each of the methods (*Naive*, *Variability corrected*, and *Full*) but not found to be associated according to the *“oracle”* method, an indication of the FDP of each method. The second column shows the proportion of associated SNPs found by each of the methods and also by the *“oracle”* method, an indication of the power of each method. Note that the parameter $n_{r}$ has no effect on the *Full* method, and differences seen are due to randomization in the sample taken. Based on 1000 iterations.

To assess validity, we repeatedly sampled $n_{r}\in \left\{500,1500,5000\right\}$ and $n_{o}=50,000$ observations to serve as our reference and original study. The results are reported compared to the complete data containing 380,776 observations, which we refer to as the *“oracle”* method. Three methods were compared: the *Naive*, *Variability corrected (Empirical)*, and the *Full*. The BH procedure at level 0.05 was applied to the 33 *p*‐values computed by each method. Using the entire data, 20 SNPs are found to be associated with the phenotype, suggesting that the actual FDR may be less than 0.05 for the various methods (since the actual FDR level of the BH procedure is typically lower than the nominal level when the fraction of non‐null hypotheses is large). The explained variance using the entire dataset is estimated to be 0.0054. All results are compared to the *“oracle”* method, utilizing all available observations (see Table 1).

The suspected false discoveries of each method are the SNPs found significant by the method in question but not by the *“oracle”* method. The average proportion of suspected false discoveries is the highest for the *Naive* method. The average proportion of false discoveries decreases as $n_{r}$ increases, but still, for $n_{r}=5000$ this proportion is higher by 50% compared to the *Full* method, serving as our baseline. The *Variability‐corrected* method is conservative, discovering on average only 8.6% of SNPs discovered by the *“oracle”* method when $n_{r}=500$. But as $n_{r}$ increases, the proportion of discovered SNPs by the *Variability‐corrected* method increases considerably.

In many realistic settings, it is expected that only a few SNPs in a region will be associated with the phenotype. In such cases, when the number of observations in the reference sample is sufficiently large, the power of the *Variability‐corrected* method is on par with the *Full* method. In the example here, the proportion of non‐null coefficients is large (recall that 20 SNPs were discovered with the *“oracle”* method), resulting in decreased power of the *Variability‐corrected* method compared to the *Full* method (see simulation in Section 5.2 for additional examples). Further investigation of the relationship between power and proportion of non‐null coefficients is explored in Section S4.3. Specifically, in Figure S4.3, we show the effect of the density of the signal on the power of the procedure. The power of the *Variability‐corrected* method is lower than that of the *Full* method, and the gap is larger in denser settings (for the same h).

## 7 Discussion

Our analysis highlights the need for variance adjustment when estimating the joint effect of SNPs from summary‐level statistics using $X_{r}$ as a replacement for $X_{o}$. We evaluate the magnitude of the increase in variance, and how it relates to the sample sizes of the reference panel and original study samples. Furthermore, we propose a method for achieving this adjustment. A significant limitation of our proposed method is its computational complexity, which scales as a polynomial of degree four in the number of SNPs, denoted by p. This complexity arises from the estimation of the sample covariance matrix variance, $V_{\hat{R}}$, which without any assumption has a complexity of $O(n_{r}p^{4})$. If $X^{*}$ can be assumed to be Gaussian, then the variance of the estimated covariance matrix has a simple expression, which reduces the computation time to $O(n_{r}p^{2})$. Otherwise, one can use a threshold estimator of β so if many entries are zero, computing ${\hat{V}}_{\hat{R}}$ has lower complexity. Additionally, parallelization can be applied to the estimation of $V_{\hat{R}}$ for faster computation. Computation time can be further reduced by using only a subset of the observations for estimating $V_{\hat{R}}$, albeit at the cost of increased variability of the estimate.

An important observation arising from our work is that the additional variance correction needed when using reference panels is governed by the ratio between the original and reference sample sizes. Our results demonstrate that even as both sample sizes increase, the extra variability introduced by estimating the covariance from the reference panel remains nonnegligible. In other words, the variance underestimation problem persists regardless of how large the sample sizes become, emphasizing that the issue is inherent to the approach rather than a consequence of limited data. The main practical benefit of Theorem 3.1 is to highlight that a fundamental problem exists in current frequentist fine‐mapping practices using summary statistics, one that is not mitigated by merely increasing sample sizes. Addressing this issue in high‐dimensional settings remains a topic for further research, with the goal of developing scalable algorithms that preserve valid inference when a large number of genetic variants is considered.

Although the current applicability of the result is limited in terms of the number of genetic variants that can be considered, we can neverthelessprovide whole genome fine‐mapping inference on computationally feasible regions using PSAT. Following marginal screening, the analyst can fix the region around the selected locations based on their computational constraint. The fine‐mapping will then provide far more information than marginal screening in that location, but less information than with an analysis on a larger neighborhood that has more than a few dozen SNPs. Specifically, we envision a table of results where each row contains the identity of an SNP that passed marginal screening, as well as the identity of the SNPs discovered in the subsequent fine‐mapping in that region. We believe that the proposed PSAT procedure, with a limited neighborhood size for fine‐mapping, is thus a useful addition to realistic GWAS analyses.

Our method has been developed for a continuous phenotype. Generalizing it to the binary phenotype is not straightforward. The covariance of the coefficients in the logistic regression model depends on the phenotype probability of each individual. These probabilities cannot be estimated using only the marginal regression summary statistics.

Another interesting application of our method is high‐dimensional meta‐analysis, where more than one study is involved. In such a case, the application of the method is straightforward (for continuous phenotypes), as long as there is an appropriate reference panel for each study population. For example, appropriate reference panels are available to meta‐analysis genomic studies on northern European and Chinese populations. Applying our proposed method will reduce the number of false discoveries compared to using a naive approach. Furthermore, the problem of sharing genotype‐level data in meta‐analysis is exacerbated as more individuals are involved from a larger number of centers.

## Conflicts of Interest

The authors declare no conflicts of interest.

## Open Research Badges

This article has earned an Open Data badge for making publicly available the digitally‐shareable data necessary to reproduce the reported results. The data is available in the Supporting Information section.

This article has earned an open data badge “**Reproducible Research**” for making publicly available the code necessary to reproduce the reported results. The results reported in this article were reproduced partially due to OS‐related issues.

## Supporting information


**Supporting File 1:** bimj70114‐sup‐0001‐SuppMat.pdf.

**Supporting File 2:** bimj70114‐sup‐0002‐Data.zip.

## Data Availability

The main packages used are available via GitHub: github.com/tfrostig/simulation_inferring_marg_association contains the code to replicate the simulation results. github.com/tfrostig/PSAT contains the code for PSAT and github.com/tfrostig/ECCCM contains the code for evaluating the covariance matrix using the reference sample.
