# Supplementary material for: Inferring on Joint Associations From Marginal Associations and a Reference Sample
Source: Biom J. 2026 Apr 13;68:e70114. doi: 10.1002/bimj.70114 (PMC13071553; doi:10.1002/bimj.70114)
Supplement: Supplementary file 1 — Supporting File 1: bimj70114‐sup‐0001‐SuppMat.pdf. [file BIMJ-68-e70114-s001.pdf]

Web-based supporting materials for: Inferring on joint associations from marginal associations and a reference sample

S1 Proof of Theorem 3.1

We aim to estimate the distribution of $\hat{\beta}_{mc} = \frac{n_r}{n_o}(X_r'X_r)^{-1}X_o'y_o$. Where X_r, X_o and y_o are standardized. Assume that $X^*_{1,.}, X^*_{2,.}, \dots, X^*_{n_o+n_r,.}$ are sampled from a distribution with expected value, $\mathbb{E}(X_i) = \mu$, and variance $\mathbb{V}(X_i) = \Sigma$, and it has a bounded fourth moment. So with out loss of generality that the first n_o observation consist of X^*_o and the last n_r observation form X^*_r. Let $\hat{\Sigma}_k = n_k^{-1}\sum_{i=1}^{n_k}(X_{k,i} - \bar{X}_k)(X_{k,i} - \bar{X}_k)'$. Define $X_{k,i,.} = d(\hat{\Sigma}_k)^{-0.5}(X^*_{k,i,.} - \bar{X}^*_k),\ k = \{o, r\}$.

From the law of large numbers as $n_k \to \infty$, then $\bar{X}^* \xrightarrow{a.s} \mu$, and $\hat{\Sigma}_k \xrightarrow{a.s} \Sigma,\ k \in \{o, r\}$. According to Slutskys' theorem

$$X_{i,.} \xrightarrow{d} d(\Sigma)^{-0.5}(X^*_{i,.} - \mu).$$

Before we turn to prove Theorem 3.1, we provide some necessary background.

Since the proof involve estimating the variance of sample covariance matrices we utilize matrix operators and transformations, which are briefly described here. We follow the same notations as in Neudecker and Wesselman (1990): vec is the vectorization operator: $vec\,(K)$ concatenates the columns of matrix $A \in \mathbb{R}^{m\times n}$ to a vector $vec(A) \in \mathbb{R}^{mn}$. Let $C \in \mathbb{R}^{p\times q}$. The Kronecker product, $\otimes$, is defined as follows $A \otimes C$ is a matrix of size $mp \times nq$ of the following form,

$$A \otimes C = [a_{i,j}C] = \begin{bmatrix} a_{1,1}C & \dots & a_{1,n}C \\ \vdots & \ddots & \vdots \\ a_{m,1}C & \dots & a_{m,n}C \end{bmatrix},$$

where $a_{i,j}$ is the (i, j) entry of A.

A useful result that combines vec and $\otimes$ is

$$vec(ABC) = (C' \otimes A)vec(B) = (C'B' \otimes I_m)vec(A), \tag{1}$$

where $B \in \mathbb{R}^{n\times p}$. Another useful property of the Kroneker product is the mixed product property for matrices A, C and B, D of suitable dimensions,

$$(A \otimes B)(C \otimes D) = (AC) \otimes (BD). \tag{2}$$

The variance of $\sqrt{n_r}\hat{R}_r$, $\mathbf{V}_{\hat{R}}$, is given in Theorem 2 of Neudecker and Wesselman (1990), reproduced here, due to its importance for our theorem.

Theorem S1.1 *[Theroem 2 - Neudecker and Wesselman (1990)] Let $\{X^*_{i,.} : i = 1, \dots n\}$ be a set of n i.i.d random vectors with correlation matrix R, covariance matrix Σ and sample covariance and correlation matrices $\hat{\Sigma}$ and $\hat{R}$. Then,*

$$\lim_{n\to\infty} \sqrt{n} \cdot vec(\hat{R} - R) \stackrel{d}{=} N_{p^2}\big(0, \mathbf{V}_{\hat{R}}\big),$$

where

$$\begin{aligned}\mathbf{V}_{\hat{R}} = \big(I - M_s(I \otimes R)M_d\big)\left(d(\Sigma)^{-0.5} \otimes d(\Sigma)^{-0.5}\right)\mathbf{V}_{\hat{\Sigma}} \\ \left(d(\Sigma)^{-0.5} \otimes d(\Sigma)^{-0.5}\right)\big(I - M_d(I \otimes R)M_s\big).\end{aligned} \tag{3}$$

$\mathbf{V}_{\hat{\Sigma}}$ *is defined as*

$$\mathbf{V}_{\hat{\Sigma}} = \mathbb{E}\left((X_{i,.} - \mu)(X_{i,.} - \mu)' \otimes (X_{i,.} - \mu)(X_{i,.} - \mu)'\right) - vec(\Sigma)vec(\Sigma)',$$

and $M_s = 0.5(I + U)$*,* $U = \sum_{i=1}^{p}\sum_{j=1}^{p} E_{i,j} \otimes E_{j,i}$ *being the permutation matrix and* $M_d = \sum_{i=1}^{p}(E_{i,i} \otimes E_{i,i})$*.* $E_{i,j}$ *is a* $p \times p$ *matrix with 1 in the* $i, j'th$ *position and zero elsewhere.*

Using the Theorem, we can derive the asymptotic distribution of the inverse correlation matrix, given in the following lemma.

Lemma S1.2 *Under the same conditions of Theorem S1.1. Then,*

$$\lim_{n\to\infty} \sqrt{n} \cdot vec(\hat{R}^{-1} - R^{-1}) \sim N_{p^2}\left(0, (R^{-1} \otimes R^{-1})\mathbf{V}_{\hat{R}}(R^{-1} \otimes R^{-1})\right). \tag{4}$$

The proof is given in the §S1.2.

We can now turn to prove Theorem 3.1, we begin by finding the expectation and variance of

$$\hat{\beta}_{mc} = n_o^{-1}\hat{R}_r^{-1}X_o'(X_o\beta + \epsilon) = \hat{R}_r^{-1}\hat{R}_o\beta + n_o^{-1}\hat{R}_r^{-1}X_o'\epsilon. \tag{5}$$

The variance is,

$$\begin{aligned}
\mathbb{V}\left(\hat{\beta}_{mc}\right) &= \mathbb{V}\left(\hat{R}_r^{-1}\hat{R}_o\beta + \frac{1}{n_o}\hat{R}_r^{-1}X_o\epsilon\right) \\
&= \mathbb{E}\left(\mathbb{V}\left(\hat{R}_r^{-1}\hat{R}_o\beta + \frac{1}{n_o}\hat{R}_r^{-1}X_o'\epsilon|X_o, X_r\right)\right) \\
&+ \mathbb{V}\left(\mathbb{E}\left(\hat{R}_r^{-1}\hat{R}_o\beta + \frac{1}{n_o}\hat{R}_r^{-1}X_o'\epsilon|X_o, X_r\right)\right) \\
&= \mathbb{E}\left(\frac{1}{n_o^2}\hat{R}_r^{-1}X_o'X_o\hat{R}_r^{-1}\sigma^2\right) + \mathbb{V}\left(\hat{R}_r^{-1}\hat{R}_o\beta\right) \\
&= \frac{\sigma^2}{n_o}R^{-1} + \mathbb{V}\left(\hat{R}_r^{-1}\hat{R}_o\beta\right) + O\left(\frac{1}{n_o n_r}\right).
\end{aligned} \tag{6}$$

The second equality follow from the law of total variance, and the last equality follows from Lemma S1.3.

Lemma S1.3 *Following the assumptions of Thereom 3.1.*

$$\mathbb{E}\left(\frac{1}{n_o^2}\hat{R}_r^{-1}X_o'X_o\hat{R}_r^{-1}\sigma^2\right) = \frac{\sigma^2}{n_o}R^{-1} + O\left(\frac{1}{n_o n_r}\right)$$

See proof in Section S1.1.

To simplify $\mathbb{V}\left(\hat{R}_r^{-1}\hat{R}_o\beta\right)$, we decompose $\hat{R}_r^{-1}\hat{R}_o\beta$ as follows:

$$\begin{aligned}
&\hat{R}_r^{-1}\hat{R}_o\beta = \left(\hat{R}_r^{-1} - R^{-1} + R^{-1}\right)\left(\hat{R}_o - R + R\right)\beta = \\
&\left(\hat{R}_r^{-1} - R^{-1}\right)\left(\hat{R}_o - R\right)\beta + \left(\hat{R}_r^{-1} - R^{-1}\right)R\beta + R^{-1}\left(\hat{R}_o - R\right)\beta + \beta.
\end{aligned} \tag{7}$$

© 0 WILEY-VCH Verlag GmbH & Co. KGaA, Weinheim

Since $\hat{R}_r$ and $\hat{R}_o$ are consistent estimators of R, and R is invertible, $\left(\hat{R}_r^{-1} - R^{-1}\right)\left(\hat{R}_o - R\right)\beta$ is a magnitude smaller than $\left(\hat{R}_r^{-1} - R^{-1}\right)R\beta$ and $R^{-1}\left(\hat{R}_o - R\right)\beta$. Note, the covariance terms involving $\left(\hat{R}_r^{-1} - R^{-1}\right)\left(\hat{R}_o - R\right)$, are also $O\left(\frac{1}{n_o n_r}\right)$. For ease of notation we define $D_r = \hat{R}_r^{-1} - R^{-1}$ and $D_o = (\hat{R}_o - R)$. We show this for $Cov(D_r D_o\beta, R^{-1}D_o\beta)$:

$$\begin{aligned} Cov(D_r D_o\beta, R^{-1}D_o\beta) &= \mathbb{E}(D_r D_o \beta\beta' R^{-1} D_o) - \mathbb{E}(D_r D_o\beta)\mathbb{E}(R^{-1}M_o\beta)' \\ &= \mathbb{E}(D_r)\mathbb{E}(D_o\beta\beta' D_o) - \mathbb{E}(D_r)E(D_o)\beta\beta'\mathbb{E}(D_o)R^{-1} \\ &= O\left(\frac{1}{n_o n_r}\right). \end{aligned} \tag{8}$$

The second equality is due to independence of D_r and D_o, the third is since $\mathbb{E}(D_o\beta) = O\left(\frac{1}{n_o}\right)$ and $\mathbb{E}(D_r) = O\left(\frac{1}{n_r}\right)$, and $\mathbb{E}(D_o\beta\beta' D_o) = \mathbb{V}(D_o\beta) = O\left(\frac{1}{n_o}\right)$.

Similar arguments can be employed to the other covariance term, $cov(M_r M_o\beta, M_r R^{-1})$ yielding the result. Due to $\hat{R}_r$ and $\hat{R}_o$ being independent, according to Eq. (7):

$$\mathbb{V}(\hat{R}_r^{-1}\hat{R}_o\beta) = \mathbb{V}\left(\hat{R}_r^{-1}R\beta\right) + \mathbb{V}\left(R^{-1}\hat{R}_o\beta\right) + O\left(\frac{1}{n_o n_r}\right). \tag{9}$$

We shall approximate the two terms in the right hand side of Eq. (9), as follows. We begin with the variance of $\hat{R}_r^{-1}$, for which the asymptotic distribution is given in Lemma S1.2,

$$\lim_{n_r\to\infty} \sqrt{n_r}\cdot vec(\hat{R}_r^{-1} - R^{-1}) \sim N_{p^2}\left(0, (R^{-1}\otimes R^{-1})\mathbf{V}_{\hat{R}}(R^{-1}\otimes R^{-1})\right). \tag{10}$$

Therefore,

$$\begin{aligned} \lim_{n_r\to\infty} n_r \mathbb{V}\left(\hat{R}_r^{-1}R\beta\right) &= \lim_{n_r\to\infty} n_r \mathbb{V}\left[vec(\hat{R}_r^{-1}R\beta)\right] \\ &= \lim_{n_r\to\infty} n_r \mathbb{V}\left[(\beta' R'\otimes I)vec(\hat{R}_r^{-1})\right] \\ &= \lim_{n_r\to\infty} n_r (\beta' R\otimes I)\mathbb{V}(vec(\hat{R}_r^{-1}))(R\beta\otimes I) \\ &= (\beta' R\otimes I)(R^{-1}\otimes R^{-1})\mathbf{V}_{\hat{R}}(R^{-1}\otimes R^{-1})(R\beta\otimes I) \\ &= (\beta'\otimes R^{-1})\mathbf{V}_{\hat{R}}(\beta\otimes R^{-1}). \end{aligned}$$

The second equality follows from Eq. (1), the fourth from Eq. (10) and the last from the mixed product property (Eq. (2)). We obtain,

$$\lim_{n_r\to\infty} \sqrt{n_r}(\hat{R}_r^{-1}R\beta - \beta) \to N_p\left(0, (\beta'\otimes R^{-1})\mathbf{V}_{\hat{R}}(\beta\otimes R^{-1})\right) \tag{11}$$

The second term, $R^{-1}\hat{R}_o\beta$ asymptotic distribution is,

$$\lim_{n_o\to\infty} \sqrt{n_o}(R^{-1}\hat{R}_o\beta - \beta) \to N_p\left(0, (\beta'\otimes R^{-1})\mathbf{V}_{\hat{R}}(\beta\otimes R^{-1})\right), \tag{12}$$

© 0 WILEY-VCH Verlag GmbH & Co. KGaA, Weinheim

since the asymptotic variance is,

$$\lim_{n_o\to\infty} n_o \mathbb{V}\left(R^{-1}\hat{R}_o\beta\right) = \lim_{n_o\to\infty} n_o \mathbb{V}\left((\beta'\otimes R^{-1})vec(\hat{R}_o)\right)$$
$$= (\beta'\otimes R^{-1})\mathbf{V}_{\hat{R}}(\beta\otimes R^{-1}),$$

where the first and second equalities are due to Eq. (1) and Eq. (12) respectively.

Now we can find the asymptotic variance of $\hat{\beta}_{mc}$,

$$\begin{aligned}\lim_{n_r,n_o\to\infty} n_r\mathbb{V}(\hat{\beta}_{mc}) &= \lim_{n_r,n_o\to\infty} n_r\left(\frac{\sigma^2}{n_o}R^{-1} + \mathbb{V}\left(\hat{R}_r^{-1}R\beta\right) + \mathbb{V}\left(R^{-1}\hat{R}_o\beta\right) + O\left(\frac{1}{n_rn_o}\right)\right)\\ &= \lim_{n_r,n_o\to\infty} n_r\left(\frac{\sigma^2}{n_o}R^{-1} + \left(\frac{1}{n_r}+\frac{1}{n_o}\right)(\beta'\otimes R^{-1})\mathbf{V}_{\hat{R}}(\beta\otimes R^{-1})\right).\\ &= \lim_{n_r,n_o\to\infty}\left(\frac{\sigma^2n_r}{n_o}R^{-1} + \left(1+\frac{n_r}{n_o}\right)(\beta'\otimes R^{-1})\mathbf{V}_{\hat{R}}(\beta\otimes R^{-1})\right).\end{aligned} \tag{13}$$

The first equality is due to Eq. (6) and (9), and second equality is due to Eq. (11) and Eq. (12). Assuming that $\lim_{n_r,n_o\to\infty}\frac{n_r}{n_o}=c$, then the asymptotic variance is,

$$\lim_{n_r,n_o\to\infty} n_r\mathbb{V}(\hat{\beta}_{mc}) = c\sigma^2R^{-1} + (1+c)(\beta'\otimes R^{-1})\mathbf{V}_{\hat{R}}(\beta\otimes R^{-1}). \tag{14}$$

Before giving $\hat{\beta}_{mc}$ we are left to show it is consistent,

$$\begin{aligned}\lim_{n_r,n_o\to\infty}(\hat{\beta}_{mc}) &= \lim_{n_r,n_o\to\infty}\hat{R}_r^{-1}\hat{R}_o\beta + \lim_{n_r,n_o\to\infty} n_o^{-1}\hat{R}_r^{-1}X_o'\epsilon\\ &= \lim_{n_r,n_o\to\infty}\hat{R}_r^{-1}\hat{R}_o\beta = \beta,\end{aligned} \tag{15}$$

where the second equality is due to $\mathbb{E}\left(n_o^{-1}\hat{R}_r^{-1}X_o'\epsilon\right)=0$, and the third is due to Eq. 7.

Since each element of the decomposed $\hat{\beta}_{mc}$ is asymptotically distributed as normal (see Eq. (11) and Eq. (12)) and according to Eq. (14) then,

$$\lim_{n_r,n_o\to\infty}\sqrt{n_r}(\hat{\beta}_{mc}-\beta)\sim N_p(0, c\sigma^2R^{-1}+(1+c)(\beta'\otimes R^{-1})\mathbf{V}_{\hat{R}}(\beta\otimes R^{-1})).$$

S1.1 Proof of Lemma S1.3

We will obtain the term (i,j) term of the matrix using e_i and e_j, from there deriving the entirety of the matrix.

$$\begin{aligned}\mathbb{E}\left(e_i'\hat{R}_r^{-1}\hat{R}_o\hat{R}_r^{-1}e_j\right) &= \mathbb{E}\left(\mathbb{E}\left(e_i'\hat{R}_r^{-1}\hat{R}_o\hat{R}_r^{-1}e_j|X_r\right)\right)\\ &= \mathbb{E}\left(e_i'\hat{R}_r^{-1}R\hat{R}_r^{-1}e_j\right)\\ &= \mathbb{E}\left(tr\left(e_i'\hat{R}_r^{-1}R\hat{R}_r^{-1}e_j\right)\right)\\ &= tr\left(\mathbb{E}\left(\hat{R}_r^{-1}e_je_i'\hat{R}_r^{-1}\right)R\right)\\ &= tr\left(\left(Cov\left(\hat{R}_r^{-1}e_j, e_i'\hat{R}_r^{-1}\right)+R^{-1}e_je_i'R^{-1}\right)R\right)\\ &= tr\left(Cov\left(\hat{R}_r^{-1}e_j, e_i'\hat{R}_r^{-1}\right)R\right)+\left(R^{-1}\right)_{i,j}.\end{aligned} \tag{16}$$

© 0 WILEY-VCH Verlag GmbH & Co. KGaA, Weinheim

Where e_i is a zero vector except for position i where the entry is 1. The fifth equality follow due to $\mathbb{E}(R^{-1}e_je_i'R^{-1}) = cov(R^{-1}e_j, e_i'R^{-1}) + \mathbb{E}(R^{-1}e_j)\mathbb{E}(e_i'R^{-1'})$. Since the asymptotic variance of $\hat{R}_r^{-1}$ tends to 0 at a rate of n_r^{-1} (see Eq. (10)), $Cov\left(\hat{R}_r^{-1}e_j, e_i'\hat{R}_r^{-1}\right)$ is $O(n_r^{-1})$ assuming a bounded fourth moment of X_i^*, multiplying by $\frac{\sigma^2}{n_o}$ yields the result.

S1.2 Proof of Lemma S1.2

According to Chapter 18, Eq. (12) of Magnus and Neudecker (2019),

$$dR^{-1} = -R^{-1}(\mathrm{d}R)R^{-1},$$

where d is a differential, a small quantity often used in matrix calculus to simplify derivative calculations (see same chapter for further details).

Applying vec to both sides of the equation,

$$vec(\mathrm{d}R^{-1}) = -vec(R^{-1}(\mathrm{d}R)R^{-1}) = -(R^{-1} \otimes R^{-1})vec(\mathrm{d}R),$$

the last equality follows from Eq. (1). According to Theorem 18.1 (tying differentials and derivatives) in Magnus and Neudecker (2019) and linearity of the derivative,

$$\frac{\mathrm{d}vec(R^{-1})}{\mathrm{d}vec(R)} = \frac{\partial vec(R^{-1})}{\partial vec(R)'} = -(R^{-1} \otimes R^{-1}).$$

Applying the delta method, we obtain the result,

$$\lim_{n\to\infty} \sqrt{n}vec(\hat{R}^{-1} - R^{-1}) \sim N_{p^2}\left(0, (R^{-1} \otimes R^{-1})\mathbf{V}_{\hat{R}}(R^{-1} \otimes R^{-1})\right).$$

S2 Computing an estimator of the variance

The expression in Eq. (14) needs to be estimated, to do so we replace all of the parameters with their estimators. However, the expression is still computationally complex to evaluate. In the following section we will describe the computation. We begin with Eq. 3, denote $P = \big(I - M_s(I \otimes R)M_d\big)$. We can rewrite Eq. 3 as $\mathbf{V}_R = P\mathbf{V}_{\hat{\Sigma}}P'$.

$$\begin{aligned} P = \big(I - M_s(I \otimes R)M_d\big) &= \Big(I - 0.5(I + K)(I \otimes R)\sum_{i=1}^{p}(E_{i,i} \otimes E_{i,i})\Big) \\ &= I - 0.5\left(\sum_{i=1}^{p}(E_{i,i} \otimes RE_{i,i}) - (RE_{i,i} \otimes E_{i,i})\right). \end{aligned}$$

Notice, that $RE_{i,i}$ is a matrix of all zeros except for the i'th column which is $R_{.,i}$. For each Kronecker multiplication of a two p by p matrices, we can look at an i, j'th block which is a $p \times p$ matrix, we denote this block by square brackets. That is the $A[i,j], A \in \mathbb{R}^{p^2 \times p^2}$ denotes the submatrix of the elements in rows $i \times p$ to $(i+1) \times p$ and columns $j \times p$ to $(j+1) \times p$.

$$\sum_{i=1}^{p}(E_{l,l} \otimes RE_{l,l})[i,j] = \begin{Bmatrix} 0, & i \neq j \\ RE_{i,i}, & i = j \end{Bmatrix}$$

and

$$\sum_{l=1}^{p}(RE_{l,l} \otimes E_{l,l})[i,j] = E_{j,j}RE_{i,j}.$$

© 0 WILEY-VCH Verlag GmbH & Co. KGaA, Weinheim

Thus, the matrix we are interested in can be written as,

$$P[i,j] = \begin{Bmatrix} I - 0.5RE_{i,i} - 0.5E_{i,i}RE_{i,i} & i = j \\ -0.5E_{j,j}RE_{i,j} & i \neq j \end{Bmatrix}.$$

The second term we are interested in is, $\Lambda = \left((d(\Sigma))^{-0.5} \otimes (d(\Sigma))^{-0.5}\right)][i,j]$,

$$\Lambda[i,j] = \begin{Bmatrix} \Sigma_{i,i}^{-0.5} (d(\Sigma))^{-0.5} & i = j \\ 0 & i \neq j \end{Bmatrix}.$$

The multiplication of these two sparse sub-matrices is $O(p^2)$ (Park et al., 1992), as at most, the number of non-zero elements of $P[i,j]$ and $\left((d(\Sigma))^{-0.5} \otimes (d(\Sigma))^{-0.5}\right)[i,j]$ is $2p$. The remaining matrix, $\mathbf{V}_{\hat{\Sigma}}$, can be partitioned as following

$$\mathbf{V}_{\hat{\Sigma}}[i,j] = Cov(\hat{\Sigma}_{i,.}, \hat{\Sigma}_{j,.}).$$

To deal with the expression $(\hat{\beta}' \otimes \hat{R}^{-1})$ in Eq. 14, we threshold $\hat{\beta}$ such

$$\hat{\beta}_i^* = \begin{Bmatrix} \hat{\beta}_{mc_i} & |\hat{\beta}_{mc_i}| \geq t \\ 0 & |\hat{\beta}_{mc_i}| < t \end{Bmatrix}.$$

Therefore, we rewrite the term as $(\hat{\beta}^{*'} \otimes \hat{R}_r^{-1})\hat{\mathbf{V}}_R(\hat{\beta}^{*'} \otimes \hat{R}_r^{-1})$.

$$(\hat{\beta}^{*'} \otimes \hat{R}_r^{-1})[1,j], = \begin{Bmatrix} \hat{\beta}_{mc_j}\hat{R}_r^{-1} & |\hat{\beta}_{mc_j}| \geq t \\ 0 & |\hat{\beta}_{mc_j}| < t \end{Bmatrix}.$$

t is some prespecified threshold, the Kronecker multiplication is denoted by a single index, as there are only p sub-matrices. We turn to write the expression of Eq. (14), as block matrix multiplication. Since all blocks are $p \times p$, the matrices are conformable,

$$(\hat{\beta}^{*'} \otimes \hat{R}_r^{-1})\hat{\mathbf{V}}_R(\hat{\beta}^{*'} \otimes \hat{R}_r^{-1}) = \sum_{i=1}^{p}\sum_{j=1}^{p}(\hat{\beta}^{*'} \otimes \hat{R}_r^{-1})[1,i]\hat{\mathbf{V}}_{\hat{R}}[i,j](\hat{\beta}^{*} \otimes \hat{R}_r^{-1})[j,1].$$

We assume w.l.o.g, that $\hat{\beta}^*$ has m non-zero elements that are positioned at the beginning of the vector (i.e in locations $1,\dots,m$ of the vector $\hat{\beta}^{*'}$),

$$\sum_{i=1}^{p}\sum_{j=1}^{p}(\hat{\beta}^{*'} \otimes \hat{R}_r^{-1})[1,i]\hat{\mathbf{V}}_{\hat{R}}[i,j](\hat{\beta}^{*} \otimes \hat{R}^{-1})[j,1] = \hat{R}_r^{-1}\left(\sum_{i=1}^{m}\sum_{j=1}^{m}\hat{\beta}_{mc_i}\hat{\beta}_{mc_j}\hat{\mathbf{V}}_{\hat{R}}[i,j]\right)\hat{R}_r^{-1}.$$

Thresholding reduces the amount of operation from p^2 to m^2. However, we still need to find $\hat{\mathbf{V}}_R[i,j]$, again writing as block matrix multiplication we obtain,

$$\begin{aligned}\hat{\mathbf{V}}_R[i,j] &= P\Lambda\hat{\mathbf{V}}_{\hat{\Sigma}}\Lambda P' \\ &= \sum_{l=1}^{p}\sum_{k=1}^{p}(P\Lambda)[i,l]\hat{\mathbf{V}}_{\hat{\Sigma}}[l,k](\Lambda P')[k,j].\end{aligned}$$

© 0 WILEY-VCH Verlag GmbH & Co. KGaA, Weinheim

The final computation is,

$$\hat{R}^{-1}\left(\sum_{i=1}^{m}\sum_{j=1}^{m}\hat{\beta}_{mc_i}\hat{\beta}_{mc_j}\sum_{l=1}^{p}\sum_{k=1}^{p}(P\Lambda)[i,l]\hat{\mathbf{V}}_{\hat{\Sigma}}[l,k](\Lambda P')[k,j]\right)\hat{R}^{-1}.$$

Estimating the covariance matrix of $vec\left(\hat{\Sigma}\right)$, is $o(np^4)$. The complexity of estimating the variance of $\hat{\beta}_{mc}$ is $o(m^2p^2 + np^4)$. Notice, that if X is a assumed to be normal then $\mathbb{V}\left(vec\left(\hat{\Sigma}\right)\right) = 2M_s(\Sigma \otimes \Sigma)$, since we plug in $\hat{\Sigma}$ it reduces the computation time to $o(m^2p^2 + p^4)$.

S3 Equivalence of testing marginal coefficients

In the following section we show the equivalence of testing the Tag-SNP marginal coefficient in both Full and Variability corrected methods. The PSAT framework requires the specification of the covariance matrix of $\hat{\beta}$ as well as matrix K used for the quadratic test static. Since in the *Full* method, the estimator used is $\hat{\beta}_o$ compared to $\hat{\beta}_{mc}$ one needs to show how matrix K is effected. Define $\eta^* = 0\ \forall i \neq j$, and $\eta^*_i = 1$, the Tag-SNP can be written as $\eta^*\hat{\beta}_m$.

That is the testing procedure is $\hat{\beta}'_m\eta^*\eta^{*'}\hat{\beta}_m > t$, where t is some threshold. Therefore, for the *Full* procedure we can write the testing as,

$$\hat{\beta}'_o\hat{\Sigma}_o\eta^*\eta^{*'}\hat{\Sigma}_o\hat{\beta}_o > t \tag{17}$$

and for the Empirical corrected procedure,

$$\hat{\beta}'_{mc}\hat{\Sigma}_r\eta^*\eta^{*'}\hat{\Sigma}_r\hat{\beta}_{mc} > t. \tag{18}$$

Thus, both method have the same selection method, but different estimates of the coefficients as well as covariance matrices.

S4 Additional Simulations

S4.1 Power in Gaussian setting

In Fig. S1, we show the same FDR level as in Fig. 1, but removing the *Naive* method for eligibility. All methods indeed control the FDR at the expected level of 0.05.

The power of almost all methods is similar when n_o and n_r increase. We present the scenario where $h \in \{5 \cdot 10^{-4}, 2.5 \cdot 10^{-3}, 5 \cdot 10^{-3}\}$, as in the rest the power is almost always 1. The *Naive* method does not appear since it does not maintain the expect FDR level. The *Full* method has a large advantage over the *Variability corrected (Empirical)* and *Variability corrected (Gaussian)* methods when n_o and n_r are small, which shrinks as they increase.

S4.2 1000Genome project data

The following simulation setting is similar to §5.2. A gene is analyzed only if the Tag-SNP estimated coefficient is above a certain threshold. However, the genotype is not simulated but rather taken from 1000Genome project. We compare the variance estimation methods (coupled with PSAT) performance. The gene analyzed is Lecithin–Cholesterol Acyltransferase (LCAT, rs:16:45409048 - 16:45412650 according to GRCh37). The SNPs were filtered to remove correlations higher than 0.99 and MAF less than 0.05, the genotype is taken from 1000Genome project. After filtration 5 SNPs are left, see Table S1 for the correlation matrix and MAF.

To conduct the simulation, we split the sample of $n = 2504$ individuals so 80% are in the original study ($n_o = 2003$) and 20% are in the reference panel ($n_r = 501$). The phenotype was generated artificially, with varying number of SNPs with

© 0 WILEY-VCH Verlag GmbH & Co. KGaA, Weinheim

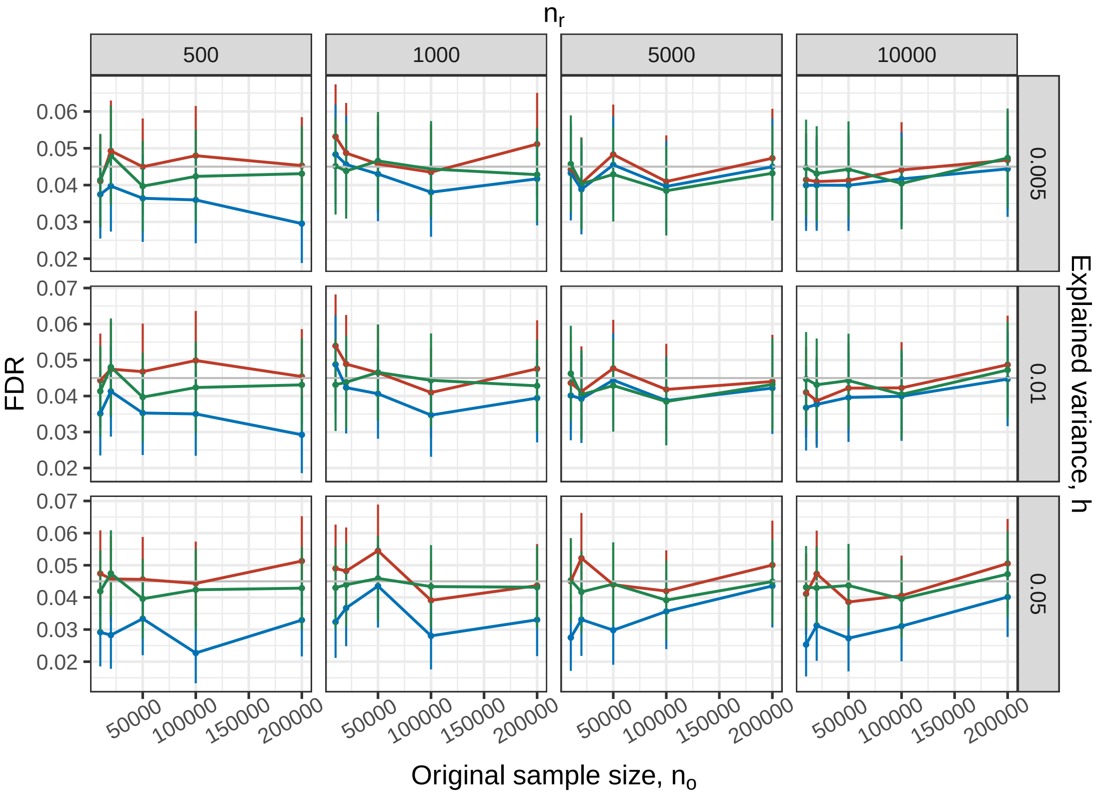

Figure S1 The power versus n_o in settings with simulated Gaussian covariates. Each plot in the facet is a combination of the reference sample size n_r (columns), and explained variance h (rows). Vertical lines represent the 2 standard error around the estimate.

Correlation	rs5923	rs13336998	rs13306496	rs1109166	rs11860115
rs5923	1.00	0.69	-0.01	0.48	0.79
rs13336998	0.69	1.00	0.01	0.36	0.88
rs13306496	-0.01	0.01	1.00	0.40	0.02
rs1109166	0.48	0.36	0.40	1.00	0.41
rs11860115	0.79	0.88	0.02	0.41	1.00
MAF	0.15	0.07	0.11	0.66	0.09

Table S1 Correlation matrix of LCAT gene. Last row is the Minor Allele Frequency.

non-zero coefficients, indexed as $\{\{1\},\{1,4\},\{3,5\}\}$, and explained variance $h \in \{0.05, 0.1, 0.15, 0.2, 0.3, 0.5, 0.75, 0.95\}$. We increase the range of h to have greater power since we are limited in the number of observations. The Tag-SNP index is 3, and the threshold is $n_o \times Z_{1-0.05}$

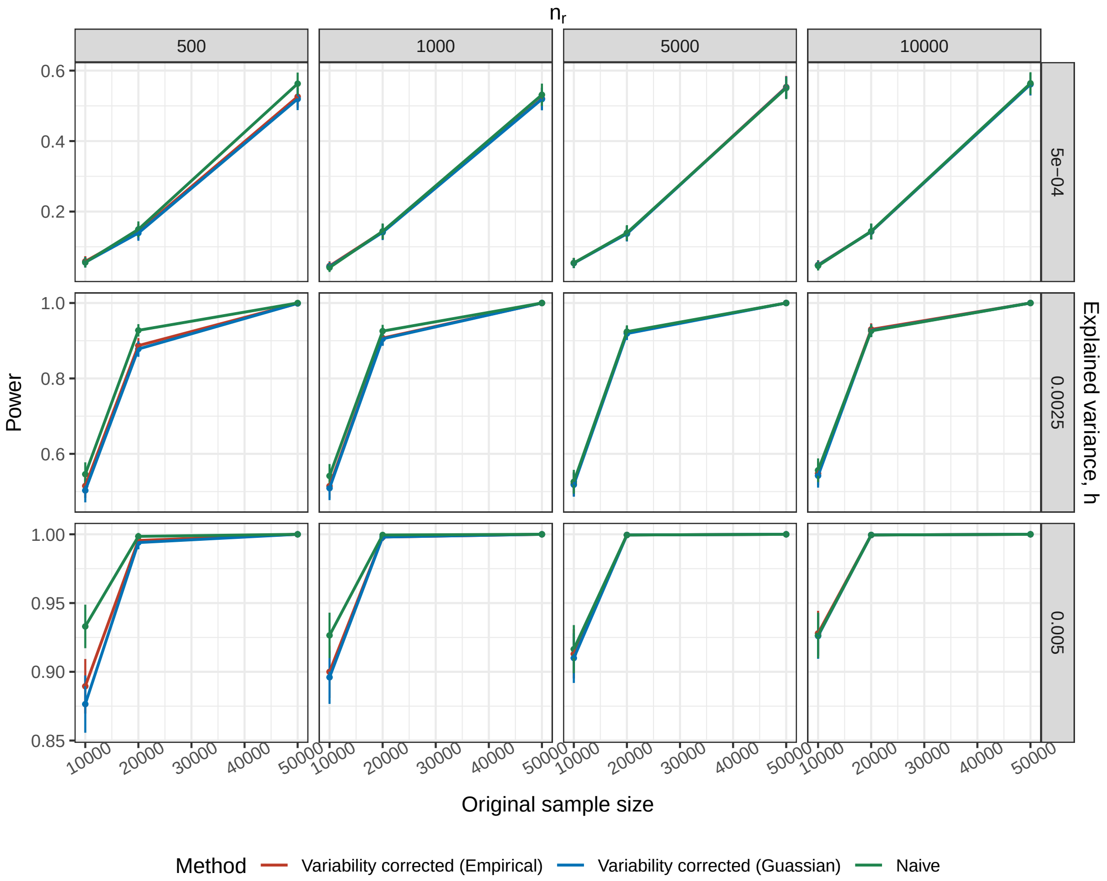

Figure S2 The power versus n_o in settings with simulated Gaussian covariates. Each plot in the facet is a combination of the reference sample size n_r (columns), and explained variance h (rows). Vertical lines represent the 2 standard error around the estimate.

Figure S3 (top row) demonstrates that correcting for selection and the reference panel covariance estimation are required to maintain the conditional FDR. In the power analysis, methods that have not maintained the conditional FDR are dropped, leaving us with the *Full*, *Variance corrected (Empirical)* and *Variance corrected (MLE)* methods.

Both the conditional and unconditional power are inspected in Figure S3. The PSAT correction becomes stricter the less likely the selection event is. When the Tag-SNP is one of the causal SNPs, $s^* = \{3, 5\}$ or the casual SNP is correlated with the Tag-SNP $s^* = \{1, 4\}$, the selection happens with probability 1. So the conditional and unconditional power curves coincide, see Figure S3 (bottom and middle rows).

In Figure S3 (bottom and middle rows), the power of the suggested method is on par with the *Full* method. As the number of causal SNPs increases (so each SNP explains less of the overall variability in the phenotype), the method's power decreases. The Tag-SNP's choice plays a crucial role in this example; when the Tag-SNP is not one of the causal SNPs (or not correlated to them, see $s^* = 1$), the power decreases substantially for all methods. There seem to be no difference in power between the *Variability corrected (Empirical)* and the *Variability corrected (MLE)*.

© 0 WILEY-VCH Verlag GmbH & Co. KGaA, Weinheim

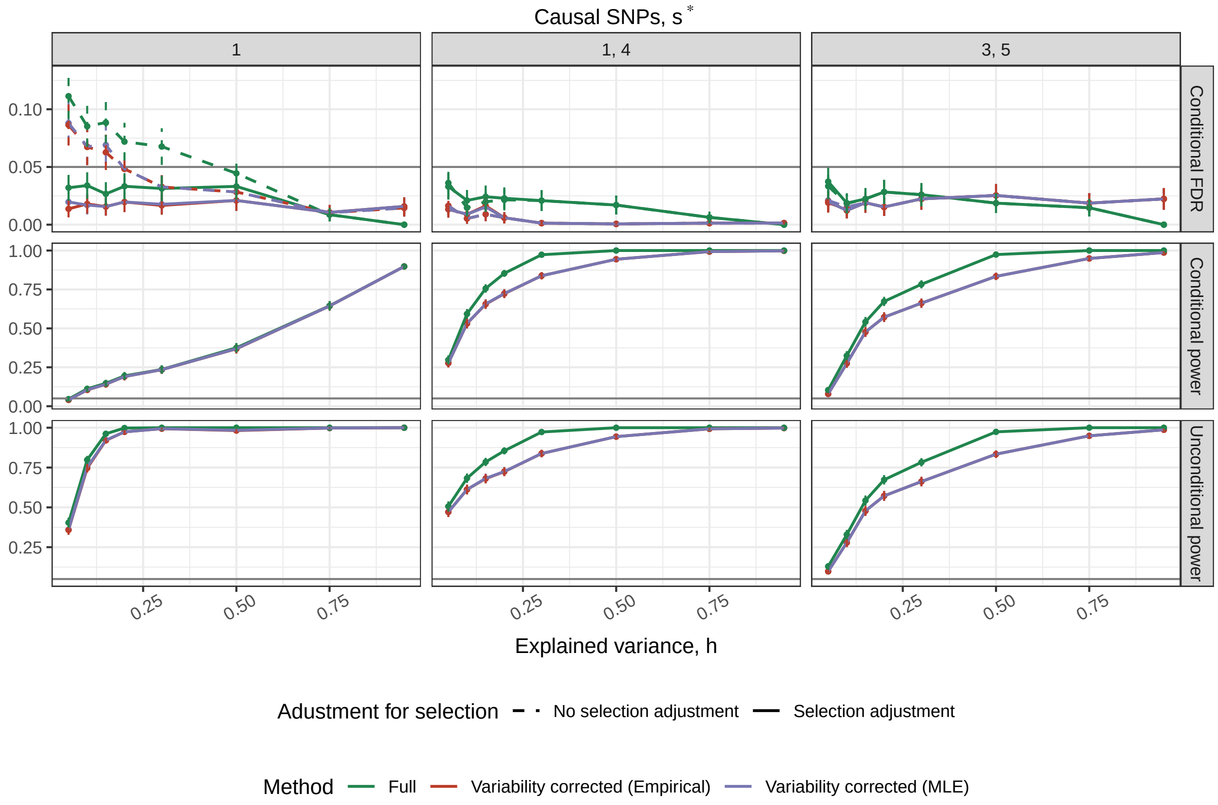

Figure S3 Results on real genetic data and simulated phenotype, using variability correction with PSAT. The columns are the set of causal SNPs, s^*, the rows are the relevant measure. The x-axis is h (Eq. (6)), $n_r = 501$ and $n_o = 2003$. The grey line passes at the expected FDR level 0.05. Vertical lines represent the 2 standard error around the estimate.

S4.3 UK-biobank simulation

In the following simulation the genomic data is taken from the UKbiobank data (Bycroft et al., 2018), while the phenotype is simulated.In order to mimic dependencies found in GWAS studies we took a single SNP found to have marginal significance on BMI (using the GIANT consortium data) on Chromsome-19. All SNPs found in the UKbiobank data in distance less than 100kb are included in the simulation.

SNPs with MAF less than 0.05 were removed, and we randomly selected pairs of SNPs to avoid correlations larger than 0.97. In total, 13 SNPs were used in the simulation. The SNPs are relatively spread across the Chromosome, leading to weaker correlations than those considered in the synthetic data simulation.

The construction of the phenotype is similar to that in the synthetic genotype simulation (§5.2). We considered $n_r \in \{500, 1500, 5000\}$, $n_o = 100,000$, and four sets of causal SNPs ($\{1, 13\}$, $\{1, 4, 9, 13\}$, $\{1, 3, 5, 7, 10, 13\}$ and $\{1, \ldots, 13\}$). We consider 3 methods, *Naive*, *Variability corrected (Empirical)* and *Full*. In this simulation the region is pre-specified (i.e., not selected from the data using marginal screening).

The results are consistent with the previous simulations finding. Figure S4 demonstrates that the *Naive* method fails in maintaining the expected FDR level of 0.05. The FDR increases with the percentage of explained variance by the SNPs, and with the ratio of n_o/n_r.

© 0 WILEY-VCH Verlag GmbH & Co. KGaA, Weinheim

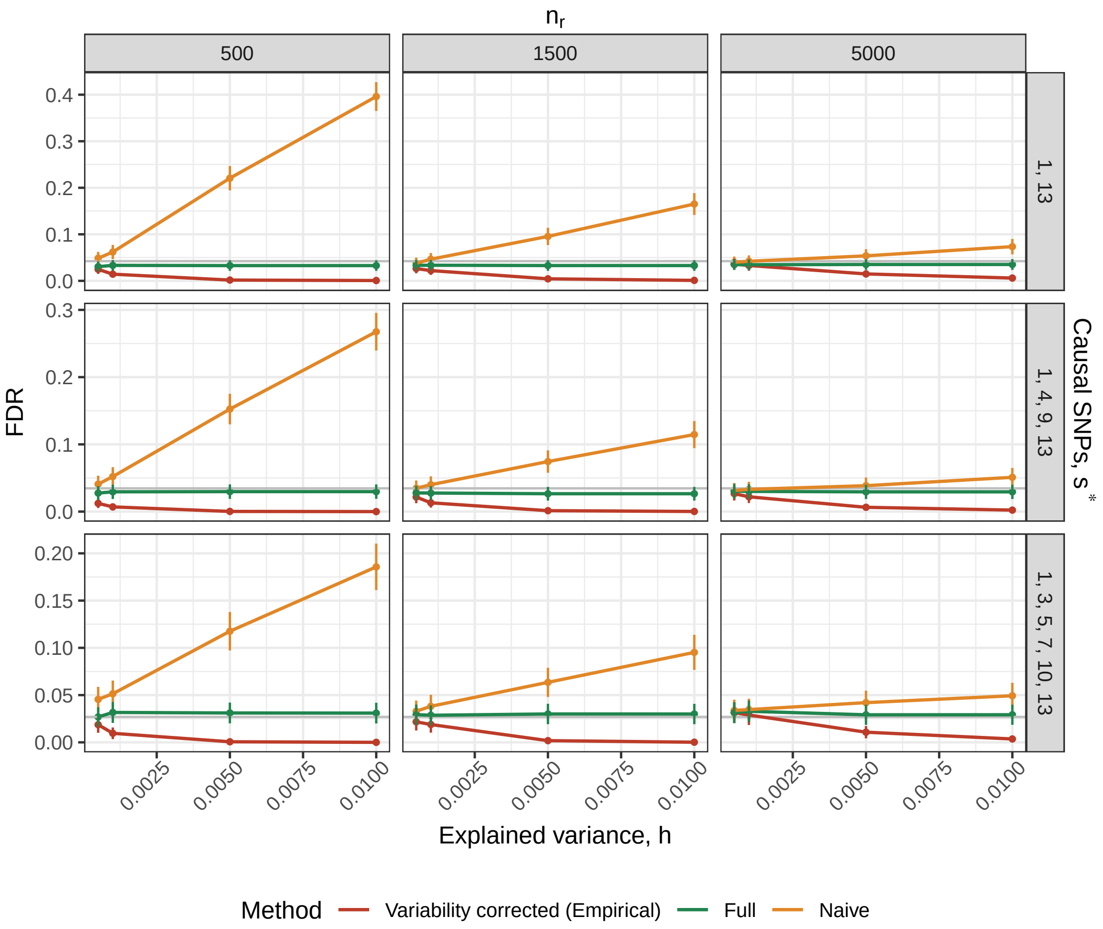

Figure S4 FDR results of UK-biobank based simulation. The rows are the set of causal SNPs, s^*, the columns are n_r. The x-axis is h (Eq. (6)) and $n_o = 100000$. Vertical lines represent the 2 standard error around the estimate.

In terms of power, the *Variability corrected (Empirical)* has similar power compare to the *Full* method in almost all scenarios. Only when n_r is small combined with a dense signal the power of the *Full* method is much higher than the *Variability corrected (Empirical)* method power. In such cases the variance inflation is similar to the magnitude of the coefficient resulting in low power. This phenomena can be seen in Fig. S5 as the non-null proportion of covariates increase the power decreases, specifically for $n_r \in \{500, 1500\}$, when the non-null proportion is 1 ($s^* = \{1, \ldots, 13\}$) the power reduces to the specified FDR level, increasing only when n_r increases to $5,000$.

S4.4 Dallas Heart Study - Application to variant selection following gene-level testing

We apply the suggested method on the data of the Dallas Heart Study (DHS) (Romeo et al., 2007). The data consists of 3549 individuals (601 Hispanic, 1830 non-Hispanic black, 1043 non-Hispanic white, and 75 other ethnicities), and four genes were considered.

The phenotype in question is Triglyceride (TG). The response variable is the residuals of log(TG) after adjusting for race, sex, and age. The data was split to original study $n_o = 3000$ and the rest of the observations were used as the reference

© 0 WILEY-VCH Verlag GmbH & Co. KGaA, Weinheim

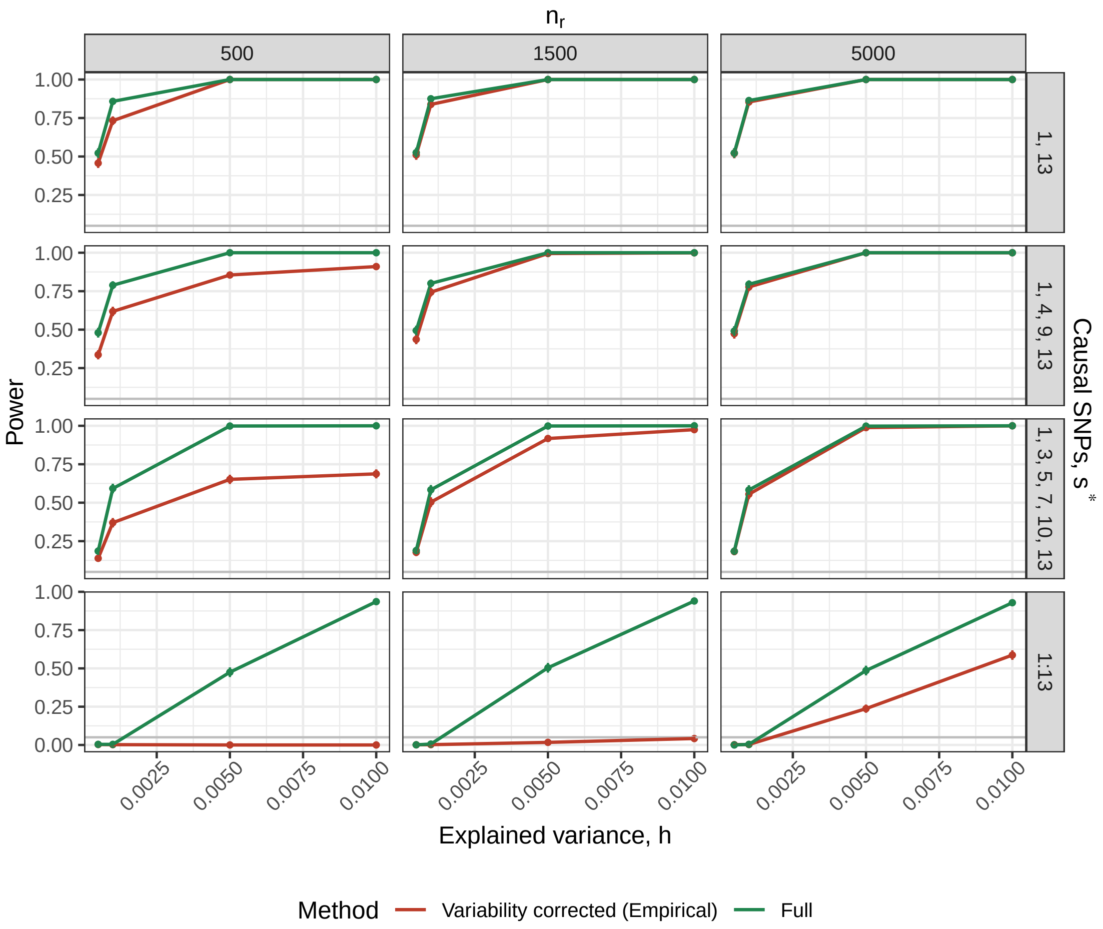

Figure S5 Power results of UK-biobank based simulation. The rows are the set of causal SNPs, s^*, the columns are n_r. The x-axis is h (Eq. (6)) and $n_o = 100,000$. Vertical lines represent the 2 standard error around the estimate.

panel, $n_r = 549$. The data split was repeated until each variant appeared at least once in the reference and original study, excluding variants that have a mutation only in a single subject.

The quadratic SKAT test (Wu et al., 2011) is used as the global-null test to select the relevant genes. As in the study of Romeo et al. (2007), four genes are tested, and only ANTPGL4 is selected if the SKAT (p-value $< 0.05/4$). After filtering the variants, we remain with 11 out of the original 32. The rest of the analysis is as described in §4. It is compared to the PSAT analysis conducted using the original data.

We present the results in Table S2. The estimated coefficients of the analysis using the summary level data and reference panel are compared with the original study analysis. The results are similar with both the coefficients and p-values. After applying BH correction, both methods find E40K, R278Q, and V308M as significant variants.

S4.5 Evaluation of Conditional Step-forward Selection

Yang et al. (2012) suggested the following conditional analysis for identifying associted SNPs across a region:

© 0 WILEY-VCH Verlag GmbH & Co. KGaA, Weinheim

	# of rare variants		Summary level data analysis		Original study analysis	
Variant	Original study	Reference panel	Estimated Coefficients	P-value	Estimated Coefficients	P-value
P5L	1	1	0.00919	0.65571	0.00814	0.61269
E40K	41	9	-0.04951	0.00381	-0.05371	0.00699
M41I	21	7	0.02572	0.17311	0.02489	0.15691
S67R	1	1	0.01478	0.45246	0.01372	0.41595
R72L	2	1	-0.00735	0.64675	-0.00837	0.68566
E190Q	24	8	0.02104	0.34591	0.01732	0.24879
T266M	1581	306	0.01687	0.24034	0.02280	0.36533
R278Q	185	22	-0.05618	0.00308	-0.05423	0.00216
V308M	2	1	0.06126	0.00075	0.06159	0.00074
R336C	6	1	0.00292	0.85407	0.00336	0.87213
G361R	1	1	0.00933	0.65040	0.00828	0.60749

Table S2 Results of analysis on 11 variants (with more than one carrier) of ANGPTL4. Column 1 and 2 show the number of variants in each sample, columns 3 and 4 the estimated coefficients according to original split and reference split and column 5,6 are the p-values. For both methods the selection was conducted using SKAT test at significance level of 0.05/4. In both methods the variants found signifcant at level 0.05 after BH correction are E40K, R278Q and V308M.

1. **Initial SNP Selection**: Begin with the most significant SNP identified from the single-SNP meta-analysis, using a strict P-value threshold (e.g., 5×10^{-8}).

2. **Conditional P-Value Calculation**: At each step, calculate the P-values of all remaining SNPs, conditional on those already selected. To avoid collinearity, if the squared multiple correlation between a candidate SNP and the selected SNPs exceeds a threshold (e.g., 0.9), the P-value for that SNP is set to 1.

3. **SNP Selection**: Select the SNP with the lowest conditional P-value, provided it is below the cutoff. If this SNP introduces collinearity with any of the selected SNPs, it is removed, and the process is repeated.

4. **Joint Model Fitting**: Fit the selected SNPs jointly in the model. If any SNP has a P-value exceeding the cutoff, it is dropped from the model.

5. **Iteration**: Repeat steps 2-4 until no further SNPs can be added or removed from the model.

This method ensures the selection of associated SNPs while managing collinearity and retaining only significant associations in the final model. The approach overcomes the problems of selection by using a stringent FWER threshold for the p-values and not updating σ as more features are incorporated into the model. Both approaches should lead to a loss of power.

While the approach takes into consideration issues caused by selective inference, the use of a reference panel still increases the type I error considerably (Fig. S6 A). To adjust for the reference panel, we used the coefficients selected, and the non-selected are considered as 0 (can be thought of as a different thresholding approach compared to 3.2). The updated approach control the error rate at the expected rate, and the power seems reasonable (Fig. S6 B).

S5 R-Package

[R-package for ECCCM routine:] R-package ECCCM containing code to obtain the variance inflation caused by using a reference panel for marginal linear regression transformation. The package can be found at https://github.com/tfrostig/ECCCM.

© 0 WILEY-VCH Verlag GmbH & Co. KGaA, Weinheim

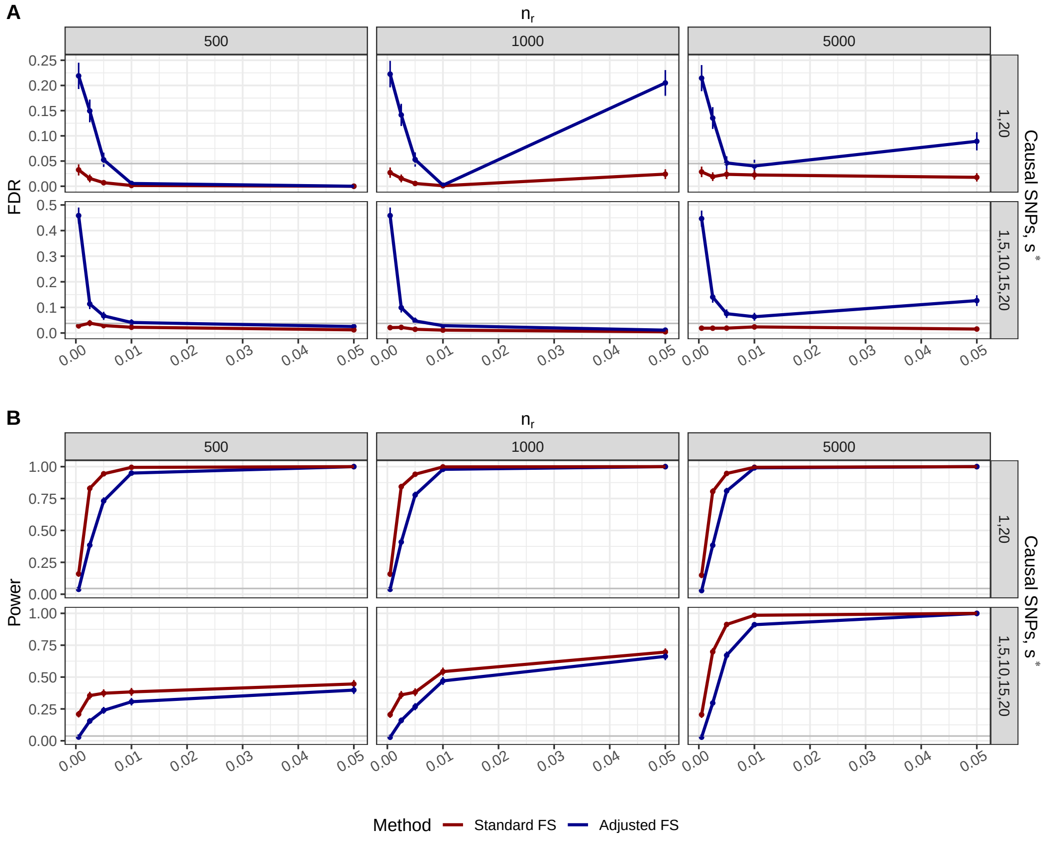

Figure S6 The FDR (A) and power (B) versus h (Eq. (6)) in settings mimicking genomic covariates. Each plot in the facet is a combination of causal SNPs indices, s^* (rows), and the reference sample size, n_r (columns). The number of observations in the original study $n_o = 10^4$ and $\rho = 0.95$ are kept constant across all scenarios.Vertical lines are the 2 standard error around the estimate and the grey horizontal line is the expected FDR level of 0.05.

References

Bycroft, C., C. Freeman, D. Petkova, G. Band, L. T. Elliott, K. Sharp, A. Motyer, D. Vukcevic, O. Delaneau, J. O'Connell, et al. (2018). The uk biobank resource with deep phenotyping and genomic data. *Nature 562*(7726), 203–209.

Magnus, J. R. and H. Neudecker (2019). *Matrix differential calculus with applications in statistics and econometrics*. John Wiley & Sons.

Neudecker, H. and A. M. Wesselman (1990). The asymptotic variance matrix of the sample correlation matrix. *Linear Algebra and its Applications 127*, 589–599.

Park, S., J. Draayer, and S.-Q. Zheng (1992). Fast sparse matrix multiplication. *Computer Physics Communications 70*(3), 557–568.

Romeo, S., L. A. Pennacchio, Y. Fu, E. Boerwinkle, A. Tybjaerg-Hansen, H. H. Hobbs, and J. C. Cohen (2007). Population-based resequencing of angptl4 uncovers variations that reduce triglycerides and increase hdl. *Nature genetics 39*(4), 513–516.

Wu, M. C., S. Lee, T. Cai, Y. Li, M. Boehnke, and X. Lin (2011). Rare-variant association testing for sequencing data with the sequence kernel association test. *The American Journal of Human Genetics 89*(1), 82–93.

© 0 WILEY-VCH Verlag GmbH & Co. KGaA, Weinheim

Yang, J., T. Ferreira, A. P. Morris, S. E. Medland, P. A. Madden, A. C. Heath, N. G. Martin, G. W. Montgomery, M. N. Weedon, R. J. Loos, et al. (2012). Conditional and joint multiple-snp analysis of gwas summary statistics identifies additional variants influencing complex traits. *Nature genetics 44*(4), 369.

© 0 WILEY-VCH Verlag GmbH & Co. KGaA, Weinheim
